# Biological early diagenesis and insolation-paced paleoproductivity signified in deep core sediment organic matter

**DOI:** 10.1038/s41598-017-01759-4

**Published:** 2017-05-08

**Authors:** Meilian Chen, Ji-Hoon Kim, Jiyoung Choi, Yun Kyung Lee, Jin Hur

**Affiliations:** 1 Department of Environment & Energy, Sejong University, Seoul, 05006 South Korea; 2 Petroleum and Marine Research Division, Korea Institute of Geoscience and Mineral Resources, 124 Gwahang-no, Yuseong-gu, Daejeon 34132 South Korea

## Abstract

The dynamics of a large stock of organic matter contained in deep sediments of marginal seas plays pivotal role in global carbon cycle, yet it is poorly constrained. Here, dissolved organic matter (DOM) in sediments was investigated for core sediment up to ~240 meters deep in the East/Japan Sea. The upper downcore profile (≤118 mbsf, or meters below seafloor) at a non-chimney site (U1) featured the exponential production of dissolved organic carbon (DOC) and optically active DOM with time in the pore water above sulfate-methane-transition-zone (SMTZ), concurrent with the increases of nutrients and alkalinity, and the reduction of sulfate. Such depth profiles signify a biological pathway of the DOM production during the early diagenesis of particulate organic matter presumably dominated by sulfate reduction. Below the SMTZ, an insolation-paced oscillation of DOM in a ~405-Kyr cycle of orbital eccentricity was observed at site U1, implying astronomically paced paleoproductivity stimulated by light availability. Furthermore, DOM dynamics of the deep sediments were likely governed by intensive humification as revealed by the less pronounced protein-like fluorescence and the lower H/C and O/C ratios below SMTZ among 15,281 formulas identified. Our findings here provide novel insights into organic matter dynamics in deep sediments.

## Introduction

As the archives of physical and biogeochemical history occurring in aquatic ecosystems, sediments in marginal seas are considered very useful in evaluating and reconstructing the paleoenvironments of both land and ocean^1–3^. There is a large stock of dissolved organic matter (DOM) in coastal sediments, playing a pivotal role in global carbon and nutrients cycling^4, 5^. Although there were numerous reports on DOM dynamics of surficial and shallow sediments, marginal sea deep sediments have received little attention, surprisingly, despite the importance.

Terrestrial- or marine-derived organic matter (i.e., allochthonous or autochthonous) undergoes many physical interactions (e.g., adsorption onto or desorption from particles), chemical reactions (e.g., redox cascade: O_2_>Mn(IV)≥NO_3_^−^>Fe(III)>SO_4_^2−^>CO_2_, abiotic geopolymerization), and microbial transformations (e.g., anaerobic sulfate reduction, methanogenesis, anaerobic oxidation of methane) once settled into sediments^5, 6^. The transformation degree of DOM in sediments is regulated by its chemical structure and lability as well as environmental factors such as temperature, redox conditions, trophic level, and microbial communities^4, 7–9^. It is believed that only <0.5% of the gross production of primary productivity on Earth can escape remineralization, being preserved in sediments^4^.

Elevated concentrations of dissolved organic carbon (DOC) and optically active DOM in sediments have been observed in pore waters, which generally serve as DOM sources to the overlying water column. It is suggested that the high level of DOM in pore waters is primarily derived from a stock of particulate organic matter (POM). It was reported that most (80–98%) POM is degraded through particulate organic carbon sulfate reduction (POCSR), and only a small fraction is affected by methanogenesis above the sulfate-methane-transition-zone (SMTZ) in non-chimney sites as opposed to the chimney sites studied in a marginal sea^10^.

Throughout the Earth’s history, some global warming events and glacial-interglacial cycles have been linked to the orbital forcing of axial precession, axial obliquity, and orbital eccentricity^11–13^. Some of these paleo-changes could be signified in the downcore profile of the pore waters. For example, pore water salinity and δ^18^O depth profiles illustrated the effects of Pleistocene glaciations^14^, and it was estimated that global average Δδ^18^O constraining the glacial-interglacial cycles is 1.0 ± 0.1‰^15, 16^. These previous findings inspired us to explore the potential effects of these paleocycles on the quantity and quality of sedimentary DOM. However, it is believed that some conditions (e.g., fast sedimentation) need to be met to maintain the non-steady-state profiles of ions to avoid potential damping-out by diffusion^17^. Moreover, the signatures of the paleo-changes in deep sediments could be hampered by clay dehydration during illitization (smectite-to-illite transformation) followed by water production (>800 mbsf) as well as by upward transport of basal fluids^18^.

Over the past decade, the fluorescent excitation-emission matrix coupled with parallel factor analysis (EEM-PARAFAC) and ultrahigh resolution Fourier transform ion cyclotron resonance mass spectrometry (FT-ICR-MS) have been proven to be among the most promising tools for characterizing and tracing DOM dynamics in sediment DOM^19–23^. While optical methods have the advantages of a high sensitivity, fast analysis, and being solvent-free without time-and labor-consuming pretreatments, FT-ICR-MS can provide molecular level DOM signature with thousands of molecular formulas. These two techniques are expected to provide complimentary information regarding the DOM dynamics in deep sediments. In the years 2007 and 2010, two deep drilling programs (UBGH1 and UBGH2) for gas hydrate were successfully performed at the Ulleung Basin (UB) in the East Sea (also known as Japan Sea), which is the eighth largest marginal sea located in the NW Pacific Ocean. Sediment cores to more than two hundreds of meters from these expeditions reflect the geological time spanning up to millions of years despite the relatively faster sedimentation rate in the studied area (average ~9–20 cm Kyr^−1^ for late Quaternary sediments)^24, 25^ than those of open oceans (average ~0.5–1.0 cm Kyr^−1^)^1, 3^. During the geological times, many environmental events, including climate paleocycles, glacio-eustatic sea level fluctuations, ocean acidification, and episodic volcanic eruptions, have reportedly occurred, potentially affecting the depositional environments of sediment^1–3, 26^.

**Figure 1:**
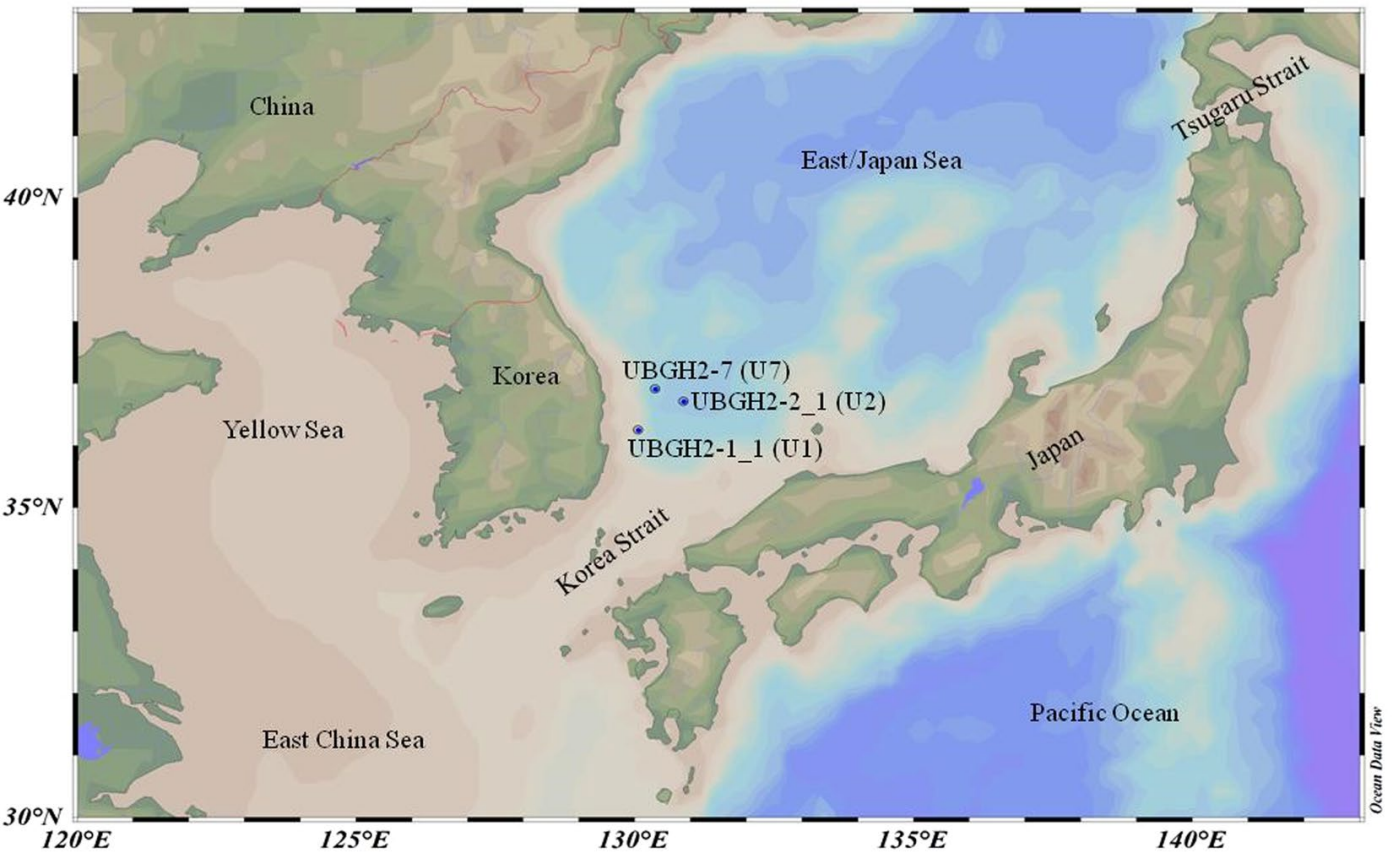
Sampling sites in the SW of the East Sea (Japan Sea) in the NW Pacific Ocean. Map produced with Ocean Data View version 4.7.8 (http://odv.awi.de)^56^.

In this study, pore waters of three deep oceanic sediment cores with different seismic characteristics were collected from the aforementioned drilling program (UBGH2) at the Ulleung Basin (Fig. 1). The main objectives of this study were three-fold: (1) to investigate the characteristics and the dynamics of DOM from shallow permeable to deeply buried oceanic sediments at different geological settings, (2) to compare the downcore trends of naturally occurring pore water DOM (PWDOM) with those of alkaline extractable organic matter (AEOM) from solid-phase sediments, and (3) to explore the paleoenvironmental changes mirrored by the sediment DOM.

## Results

### Horizontal comparison of DOC, CDOM, and FDOM in PWDOM

Chromophoric DOM (CDOM), which including fluorescent DOM (FDOM), is a pool of light-absorbing DOM, which plays various important roles in aquatic ecosystems. The core depths were 216, 192, and 238 mbsf (meters below seafloor) at sites U1, U2, and U7, respectively (Table S1). The seismic profiles of these three sites are shown in Fig. S1. As we can see from the figure, site U1 is a non-chimney site whereas site U2 is a truncated chimney site and site U7 is a chimney site. While non-chimney site U1 had an established age model of ~1.62-Myr (Table S2), the remaining sites, U2 and U7, were difficult to establish age models due to their chimney structure but estimated up to ~2.5-Myr old according to the age-depth relation in close proximity^1, 3^. As implied above, the seismic chimney can facilitate the migration of gases and fluids up through the sediments, which may exert an influence on the apparent differences of the DOM quantity and the quality of the three sites. For PWDOM, the bulk DOC and CDOM (expressed as absorption coefficient at 254 nm and 350 nm, i.e., *a*_254_ and *a*_350_) followed the order of U1 > U2 > U7 at the same depth (i.e., non-chimney >truncated chimney> chimney) (Fig. S2). The relatively lower values in the seismic chimney sites could be attributed to the upward migration of the deep fluids, which were produced during clay dehydration (i.e., dilution effect) with higher freshening ratios (up to 53%, freshening ratio = (Cl^−^_seawater_−Cl^−^_pore fluid_)/Cl^−^_seawater_ x 100) relative to <5% at non-chimney site U1 which is also demonstrated by downcore profiles of chlorinity at these sites (Fig. S3)^18^. For fluorescent DOM (FDOM) of PWDOM, three humic-like components were identified by PARAFAC modeling (Figs S4 and S5). The FDOM components followed the order of U1 > U2 > U7 at the same depth, implying potential venting/seeping of presumably bio-refractory and pre-aged PWDOM into deep ocean. Since we found that chimney sites were heavily affected by the dilution effects, we will focus below on the non-chimney site U1, which hopefully can mirror the paleoenvironmental changes more accurately.

### Downcore profiles of DOM for PWDOM and AEOM at site U1

**Figure 2:**
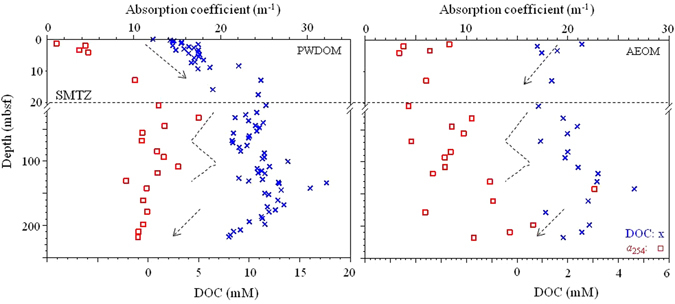
Downcore profile of DOC and CDOM of PWDOM and AEOM at non-chimney site U1. Note the top 21 m showed in amplified scale.

**Figure 3:**
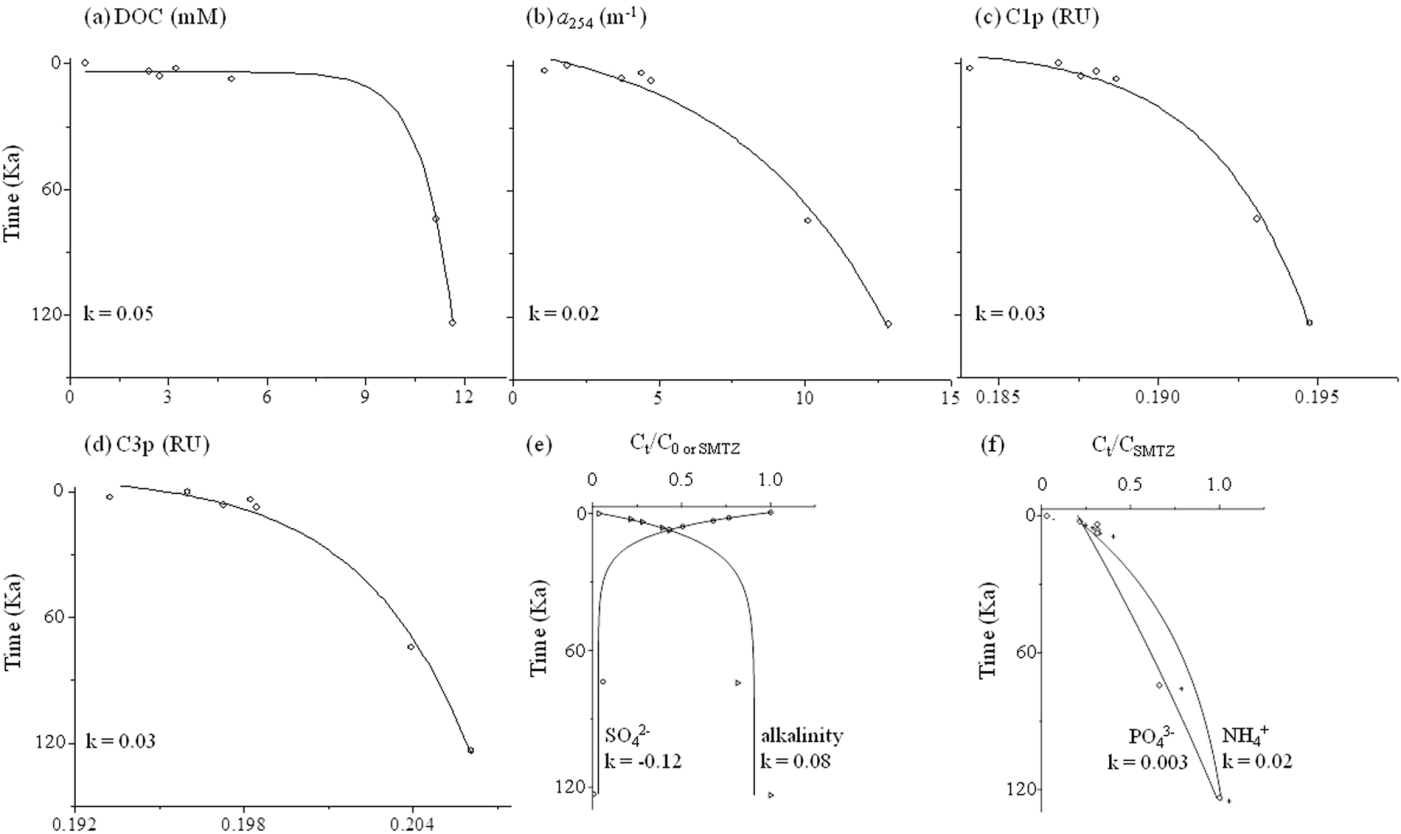
Examples of first order kinetics exponential production of DOC, CDOM, and FDOM above the SMTZ (~21 mbsf) in pore waters at site U1 (R^2^ ≥ 0.98) concurrent with the production (R^2^ ≥ 0.89) of alkalinity and nutrients (PO_4_^3−^ and NH_4_^+^) and the reduction (R^2^ = 1.00) of sulfate (SO_4_^2−^) (*p* < 0.0001). Rate constant k unit: Kyr^−1^.

**Figure 4:**
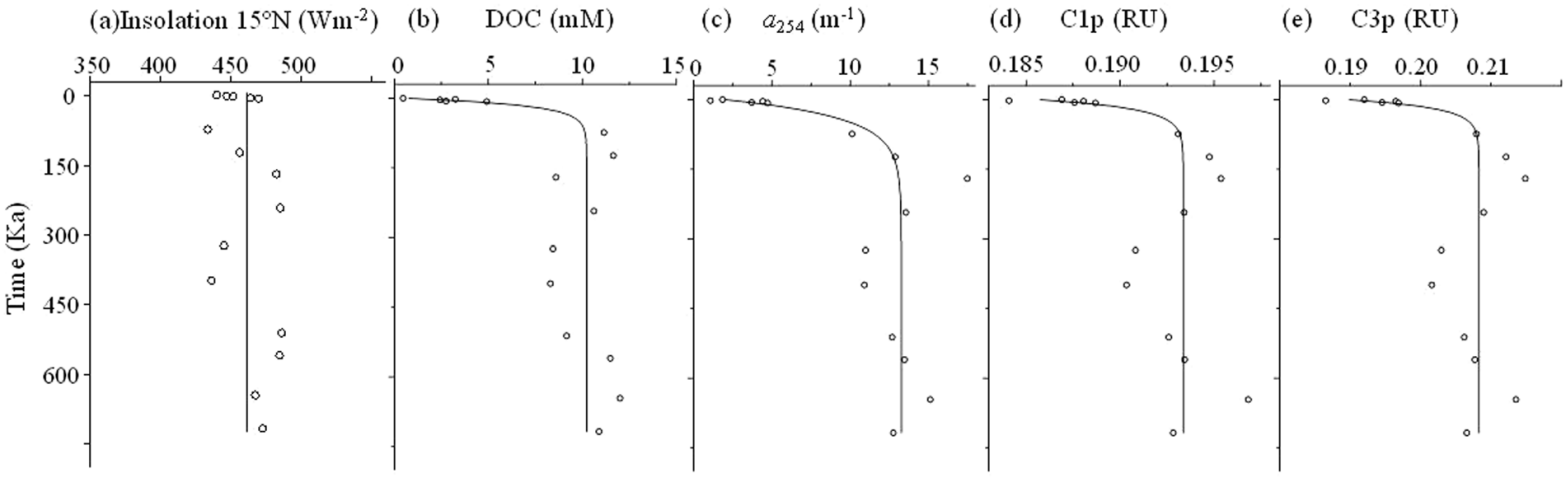
Examples of exponential production (R^2^ ≥ 0. 98, *p* < 0.0001) of DOC, CDOM, and FDOM with time above the SMTZ followed by insolation-paced oscillations (linear correlation R^2^ ≥ 0. 4, *p* < 0.05) in the downcore profiles of pore waters at site U1.

**Figure 5:**
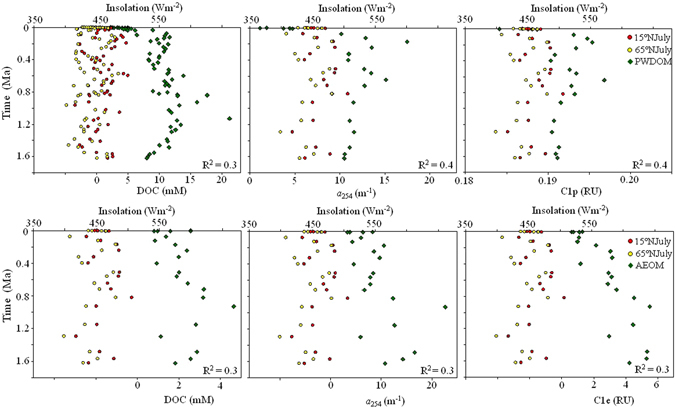
Co-variation of the insolation at 15°N and 65°N in July with PWDOM (upper row) and AEOM (lower row) parameters in a ~405-Kyr cycles at site U1 (*p* < 0.05). Linear correlation excluding the depth above the SMTZ and below 118 mbsf. Insolation and age model based on literature^1, 31^.

As seen from Figs 2–4, the DOC, CDOM, and FDOM from PWDOM generally increased exponentially with time from the seafloor to the SMTZ (~123-Ka at ~21 mbsf) at site U1, opposite the general trends of those in AEOM (Fig. 2 and Fig. S6). The depth of the SMTZ in this study was inferred from the sulfate concentration in the pore waters, which was almost zero at the depth of ~21 mbsf and coincided with the maximum depth of Group-1 (G1) after cluster analysis based on AEOM data of DOC, optical, molecular, and elemental parameters (Fig. S7). It is different from the inferred SMTZ depth of 7.7 mbsf at the same site in a previous study but rather corresponds to the expanded SMTZ^6^. The increasing DOC vertical profiles in the upper sediments were consistent with recent reports of organic matter cycling across the SMTZ in oceanic sediments^27, 28^. The rate constants k, best fit to first order kinetics (Eq. 1), ranged from 0.01 to 0.05 Kyr^−1^ with the DOC at the higher end and the terrestrial humic-like component C2p at the lower end (Table 1). The model fitting had R^2^ > 0.98 (*p* < 0.0001) except for the C2p (R^2^ = 0.3, *p* < 0.05). Below the SMTZ, the DOC, CDOM, and FDOM from PWDOM and AEOM as well as sediment total organic carbon (TOC), the molecular formula data such as intensity weighted average (wa) of m/z ratio and sulfur element number, i.e., m/z_wa_ and S_wa_, showed general cyclic variations at site U1 (Figs 4 and 5 and Figs S9–S11).

**Table 1:** Net production (+) and reduction (−) and kinetics rates (unit: Kyr^−1^) of PWDOM and pore water chemistry parameters at site U1.

Item	DOC	*a*_254_	*a*_350_	C1p^Ɨ^	C2p^Ɨ^	C3p^Ɨ^	Alkalinity	NH_4_^+^	PO_4_^3−^	SO_4_^2−^
Unit	mM	m^−1^	m^−1^	RU	RU	RU	mM	mM	µM	mM
Net production^ǂ^	11.2	11.0	0.7	0.01	0.0001	0.01	68.4	5.8	408	−26.0
k_SMTZ,1st order_	0.05	0.02	0.03	0.03	0.01	0.03	0.08	0.02	0.003	−0.12

^ǂ^The value at the SMTZ depth (~21 mbsf) minus that at the depth of zero.

For FDOM, 64 PWDOM EEMs and 21 AEOM EEMs were imported separately into MATLAB (version 7.0.4), and three FDOM components for PWDOM (C1p to C3p) and another three components for AEOM (C1e to C3e) were identified after the modeling. The contours and the excitation/emission maxima (Ex/Em) are presented in Fig. S4 and Table S3. From the spectral features of the FDOM components, C1p (Ex/Em maxima: 295/395 nm) was assigned as a marine/microbial humic-like component, and C2p (Ex/Em maxima: 340/430 nm) traditionally referred to as terrestrial humic-like components, and another humic-like C3p was found (Ex/Em maxima: 260/470 nm). For the FDOM of AEOM, C1e (Ex/Em maxima: <260(315)/420 nm), C2e ((260)390/478 nm), and C3e (295/305 nm) have been traditionally assigned as a marine/microbial humic-like, a red-shifted terrestrial humic-like, and a protein-like component, respectively^29, 30^. A detailed comparison between PWDOM and AEOM can be found in the supporting information, and we will focus on the naturally occurring PWDOM here. Although the FDOM in PWDOM was relatively stable for the three sites with the values changing within the range of 0.18–0.21 RU for all three components, it still showed site-dependent trends with depth (Fig. S4). For example, similar to the trends of DOC and *a*_350_, the absolute abundance of the PWDOM EEM-PARAFAC components displayed orbital-scale cyclic variation for site U1 below the SMTZ. In the upper sediments (<~21 mbsf), however, all the PWDOM FDOM components increased exponentially with depth (Fig. 3).

**Figure 6:**
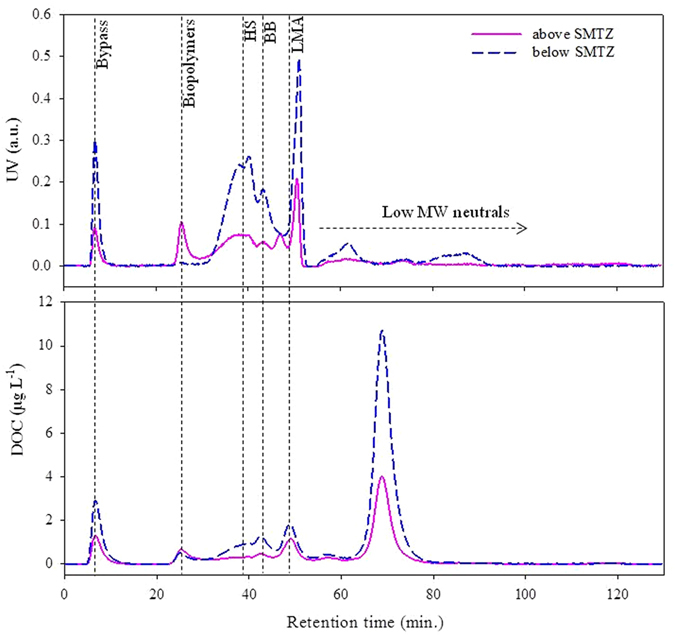
SEC-UVD (254 nm) and SEC-OCD of PWDOM above and below the SMTZ. Biopolymers ≥10 kDa. HS = humic substances; BB = building blocks; LMA = low molecular weight acids. Bypass was to obtain a detector signal at the dead volume time of each run.

For molecular weight (MW) distribution measured with size exclusion chromatography coupled with an ultraviolet detector and organic carbon detector (SEC-UVD/OCD), most of the fractions, including humic substances (HS), building blocks, low MW acids, and low MW neutrals, increased below the SMTZ except for biopolymers fraction which displayed the opposite trend (Fig. 6). The details of size fraction assignments can be found elsewhere^31, 32^. The higher MW biopolymer fraction constitutes mainly polysaccharides, proteins, or amino sugars^31^, which may be more bio-labile. For this study, optically invisible low MW neutrals fraction were abundant in PWDOM, especially below the SMTZ.

**Figure 7:**
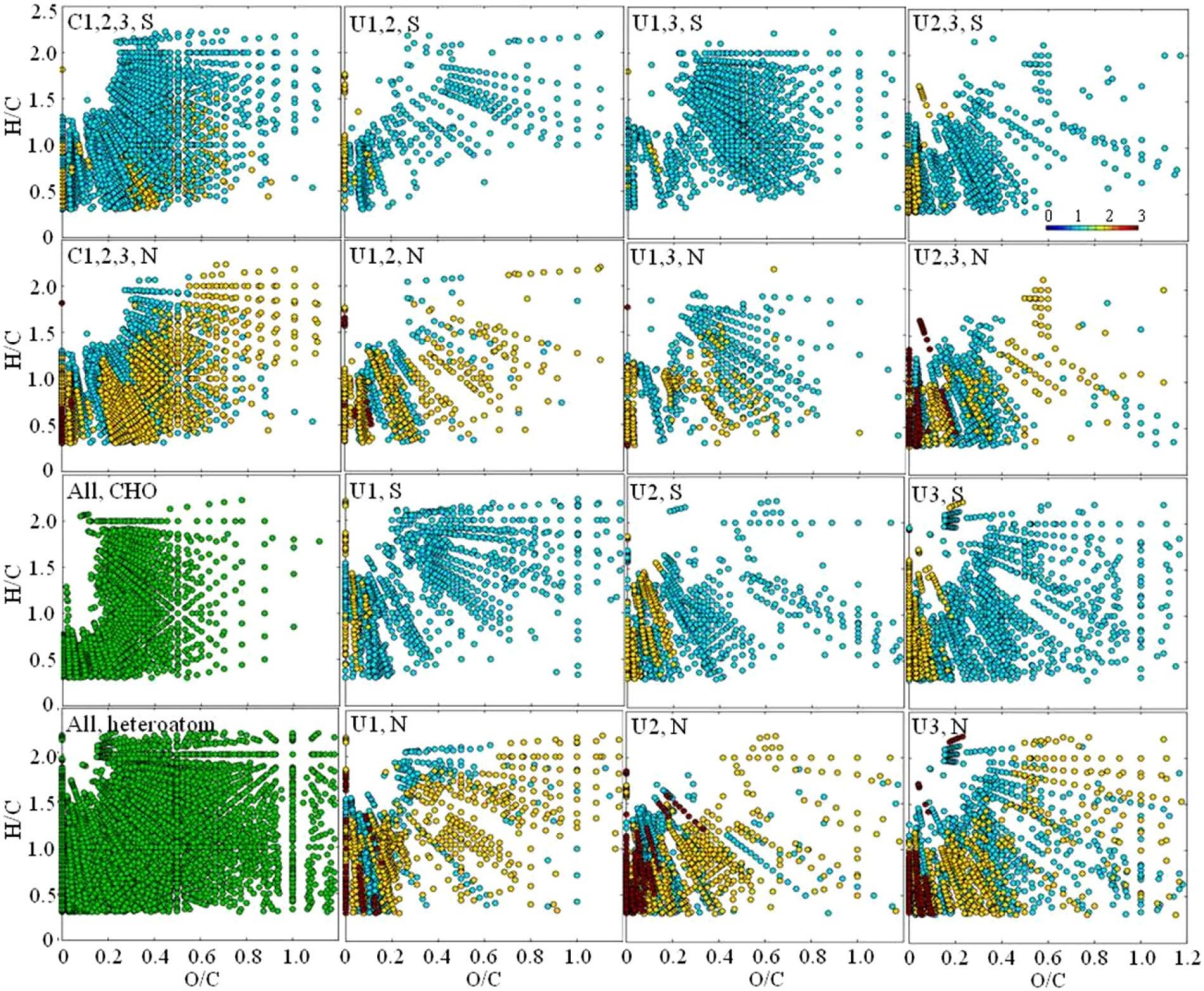
van Krevelen diagrams of all molecular formula diagrams (green) and common (C) and unique (U) heteroatomic (N and S) formula among three sub-groups (1–3) for AEOM at site U1. Light blue: 1-heteroatom; yellow: 2-heteroatom; dark red: 3-heteratom; green.

From the molecular formula data of AEOM measured with FT-ICR-MS, it was found that heteroatomic S- and N-containing formulas (58% and 65% among diverse 15,281 identified non-redundant formula) were highly enriched, occupying a wide area in the van Krevelen diagram, in contrast to a low abundance (13%) of CHO-only formulas which were primarily clustered to the left side of the van Krevelen diagram with O/C < 0.5 (Fig. 7). After a cluster analysis leading to three sub-groups (G1 to G3) distinguished by depth (G1:1.3 to 21 mbsf; G2:32 to 118 mbsf; G3: 131 to 218 mbsf, see supporting information and Fig. S7), the common and unique heteroatomic formulas in each sub-group were compared among them (Fig. 7). As seen from the figure, while common heteroatomic formulas were abundant and widely distributed, the unique formulas in G1 and G3 fell into the categories of proteins, carbohydrates, lipids, lignins/carboxyl-rich alicyclic molecules (CRAM), unsaturated hydrocarbons, and condensed aromatic structures. Meanwhile, the unique formula in G2 primarily fell into the region of condensed aromatic structures, unsaturated hydrocarbons, and lignins/CRAM. The unique formulas in both G1 and G3 appear mainly clustered in the middle part which can be assigned as lignins/CRAM and proteins.

## Discussion

As seen from Figs 2 and 3 and Fig. S6, production with time was observed above the SMTZ for DOC (Δ = 11.2 mM, Δ = value_SMTZ_ − value_depth = 0_; See Table 1), CDOM (Δ = 11.0 m^−1^ for *a*_254_ and 0.7 m^−1^ for *a*_350_), and FDOM (Δ = 0.01 RU, 0.0001 RU, and 0.01 RU for C1p, C2p, and C3p, respectively) in PWDOM (R^2^ > 0.98, *p* < 0.0001, except for C2p R^2^ = 0.3, *p* < 0.05), which were concurrent with the reduction of those in AEOM (R^2^ > 0.3, *p* < 0.05). The amount of net AEOM reduction was estimated as follows: ΔDOC = −1.7 mM, Δ*a*_254_ and Δ*a*_350_ = −4.0 m^−1^ and −2.3 m^−1^, ΔC1e, ΔC2e, and ΔC3e = −0.3 RU, −0.6 RU, and −0.1 RU, respectively. The net production and reduction amounts as well as the first-order kinetic rate constants of PWDOM production and AEOM reduction are summarized in Table 1 and Table S4.

Although exponential decrease of reactive particulate organic carbon (POC) with depth during early diagenesis was proposed decades ago by Berner^33^ and exponential-like profiles of DOC in anoxic sediments have been previously observed^5^, this is the first report on exponential production of both DOC and optically active DOM pool (i.e., CDOM and FDOM) in marginal sea sediments concurrent with AEOM reduction above the SMTZ. Production of FDOM above the SMTZ was also observed in the Arctic sediments, in which a shallow site at the Chukchi Shelf receiving large organic matter loadings featured the most pronounced production^34^. Furthermore, the exponential production of DOC, CDOM, and FDOM above the SMTZ were concomitant with the time-dependent variations of nutrients and alkalinity (i.e., exponential increasing trends) and sulfate (i.e., exponential decreasing trend) in pore waters (Fig. 3, R^2^ > 0.98, *p* < 0.0001), signifying a biological pathway of PWDOM production via particulate organic matter degradation. POCSR was reported to be a predominant organic matter degradation pathway (80–98%) above the SMTZ in non-seismic chimney site U1^10^. Net production of FDOM was found to be linearly correlated with POCSR rates in the Arctic sediments^34^. In this study, DOM (except for humic-like C2p) production was found to be reversely correlated with sulfate concentrations above the SMTZ (R^2^ > 0.80, *p* < 0.05, Table S5). Although both C2p and C3p could be assigned as traditionally termed terrestrial humic-like based on the spectral features, C3p behaved more like the marine humic-like component C1p compared to C2p, exhibiting the higher net production rate in the sediment (0.03 vs. 0.01 Kyr^−1^) and the higher correlation R^2^ with insolation (0.4 vs. 0.2). Indeed, the actual sources of these traditional termed “terrestrial” vs. “marine” humic-like components are still controversial, and the related evidences are emerging that they could actually be derived from both end members depending on ecosystems^29, 35, 36^. The optical and molecular signatures revealed more proteins and carbohydrates and higher H/C_wa_ (>1.0) and O/C_wa_ above the SMTZ (i.e., sub-group G1) relative to those below the SMTZ (Figs S7, 8 and 12, Tables S6 and S7). It is noteworthy that the AEOM parameters generally had much lower correlation coefficients when fitted into the exponential reduction kinetics, probably due to the potential preferential extraction of more aromatic structures^37^. In a recent report, a new reaction-transport model was described and the initial “depolymerization” by hydrolysis, oxidative cleavage, and others was proposed to be a rate limiting step of sediment organic matter degradation^27^. It was also believed that refractory DOM represents the majority (>95%) of the total PWDOM^27^. Therefore, it seems reasonable to observe this exponential production phenomena for bulk DOC and generally bio-refractory CDOM and FDOM in natural ecosystems^34^. It is unclear if this pattern can hold for other DOM fractions, meriting further studies. For the intensity weighted molecular formula data of AEOM, interesting, only formula element number of N_wa_ showed net reduction, potentially due to its linkage to bio-labile proteins and amino acids (Table S4).

The sediments from site U1 are very feasible for paleoceanographic reconstruction since the logging-while-drilling (LWD) gamma-ray log at this site has shown a good correlation with the “LR04” stack of global benthic δ^18^O records to establish the age-depth tie^1^. The freshening ratio is very low (<5%) as compared to up to 53% at certain other sites^18^, implying that the site is much less affected by the basal fluids upward migration. One interesting point of the depth profile at site U1 is the apparent cyclic oscillation of the DOM variables. The cyclic patterns interestingly coincided with the ~405-Kyr insolation (solar radiation) cycles (15°N and 65°N in July, the insolation at two different latitudes has a strong linear relationship with R^2^ > 0.953, *p* < 0.0001), the periodicity of the Earth’s orbit eccentricity minima (Fig. 5)^38, 39^. Although the astronomical pacing of the paleoenvironments, such as the glacial-interglacial periods and the Paleocene-Eocene thermal maximum, is already well known^12, 15, 16^, it is encouraging to observe this cyclic oscillation for a diverse of DOM parameters (except for protein-like C3e and element number of N_wa_, potentially linked to bio-labile organic matter) measured utilizing both the optical and molecular techniques. As seen from Table 2 and Table S8, the linear correlation coefficients square (R^2^) between the insolation and DOM variables ranged between 0.2–0.4 with *p* < 0.05 for PWDOM and 0.1 to 0.3 for AEOM, excluding the data above the SMTZ and below 118 mbsf, which were likely more significantly impacted by microbial or geological factors and the related changes in pore water chemistry. The relative higher degree of the correlation for PWDOM versus AEOM, especially for molecular data, could be associated with the preferential extraction and semi-quantitative nature of the FT-ICR-MS technique. Meanwhile, for the terrestrial humic-like component C2p in PWDOM and C2e in AEOM also illustrated relatively lower correlation coefficients (R^2^ = 0.2), probably due to physical factors (e.g., fluvial mobilization) involved in terrestrial DOM inputs to marginal sea sediments. No hysteresis was observed here presumably due to the negligible time for organic matter from ocean surface settling down to the sediments compared to the 405-Kyr. Although the glacial-interglacial cyclic variations of density, biogenic opal, and total organic carbon have been previously reported in this region^1, 40^, these shorter cycles could not be reflected in the data presented in this study since it needs higher resolution records. The estimated oscillations amplitude in PWDOM were 3.5 mM for DOC, 2.3 m^−1^ and 0.6 m^−1^ for *a*_254_ and *a*_350_, and 0.4 RU, 0.2 RU, and 0.4 RU for C1p, C2p, and C3p (Table 2). These values were estimated using an insolation rising phase from depths of 68 to 118 mbsf (399- to 718-Ka). In addition, it was estimated that ΔDOM/Δinsolation = 0.10 mM m^2^W^−1^ (for DOC), 0.06 mW^−1^ (for *a*_254_), and 0.0^2^ mW^−1^ (for *a*_350_) in PWDOM. The ratios of ΔDOM/Δinsolation for FDOM in pore waters were estimated to be 0.0001 RU m^2^W^−1^, 0.00003 RU m^2^W^−1^, and 0.0002 RU m^2^W^−1^ for C1p, C2p, and C3p, respectively. Further study is warranted for the patterns of other DOM fractions.

**Table 2:** Relationship between insolation (15°N in July) and DOM parameters at non-chimney site U1.

Items	DOC	*a*_254_	*a*_350_	C1p	C2p	C3p
Unit	mM	m^−1^	m^−1^	RU	RU	RU
R^2^	0.3	0.4	0.2	0.4	0.2	0.4
Amplitude^ǂ^	3.5	2.3	0.6	0.003	0.001	0.006
ΔDOM/Δinsolation*	0.10	0.06	0.02	0.0001	0.00003	0.0002

Correlation excluding the depth above the SMTZ (21 mbsf) and below 118 mbsf (affected by fluids migration) (*p* < 0.05).

^ǂ^*Estimated using an insolation rising phase from depth 68 to 118 mbsf (399- to 718-Ka). Insolation unit: Wm^−2^.

The combined results suggest that the oscillations of surface productivity paced by the insolation cycles can be a plausible scenario leading to the ~405-Kyr cycles of the DOM parameters at the non-chimney site U1. For example, higher primary productivity in the ocean and land is most likely stimulated by the higher insolation and vice versa due to light limitation. In addition, it is not surprising that the correlation coefficients were not too high because the light availability was not the sole control factor for the primary productivity. Subsequent sedimentation and diagenetic processes as well as fluvial transport (in case of terrestrial-derived DOM) may also affect the DOM finally preserved in the sediments. It is also notable that certain conditions (such as fast sedimentation >~200 cm Kyr^−1^ for small ions) need to be met for this non-steady-state profiles in pore waters to avoid the damping-out effect by diffusion^17^. For pore water DOC, CDOM, and FDOM in this study, there are several possible scenarios leading to the observed oscillation without the influence of fast sedimentation: (1) continuous release of DOM from the POM, (2) negligible diffusion due to low diffusion coefficient and/or low porosity of deep sediments, and (3) a combination of the two. For the first possibility, POM is well known to be a standing stock of DOM in the pore waters via POM degradation and/or desorption^5^. For the second possibility, diffusion flux J = sediment porosity × diffusion coefficient × concentration gradient, i.e., J = ɸ_o_D_s_(dC/dZ)_o_ according to the Fick’s first law of diffusion. With very low porosity and diffusion coefficients, the diffusion would be insignificant. The porosity is known to generally decrease with depth due to the sediments compaction, and thus the porosity is expected to be lower below the SMTZ (~0.86 at interface while ~0.68 below the SMTZ, Fig. S3)^6^. Large sized organic molecules may make contribution to the low diffusion according to the empirical relationship between MW and diffusion coefficient (i.e., log D° = 1.72–0.39 × log MW, D°: free solution diffusion coefficient at 25 °C in distilled water)^41^. From FT-ICR-MS data, we observed super-rich heteroatomic elements of S and N in the sediment organic matter (Fig. 7). Sulfur is well-known to serve as “bridge” to crosslink molecules together. In addition, humic substances of CDOM and FDOM are believed to assemble together into high apparent molecular weight (AMW, up to ~1 MDa) supramolecular geopolymers, which are stabilized predominantly by dispersive forces such as van der Waals and H-bonding^42^. Therefore, the requirements for DOM geopolymers with much bigger AMW to maintain non-steady-state downcore profiles in pore waters should be different from those of small MW ions. To confirm our inference on bigger AMW in deep sediments, we investigated the AMW using SEC-UVD and SEC-OCD; despite a dramatic increase of humic substance below the SMTZ, some low MW acids and an abundant amount of low MW neutrals were still observed in the chromatograms, respectively (Fig. 6). This observation, on one hand, seems to partly support the DOM humification with depth as also observed via optical and FT-ICR-MS data. On the other hand, it is also partly consistent with the size-reactivity continuum model that smaller size DOM fractions are less bioavailable^43^. Therefore, the observations here support the scenario of the combined effects of lower diffusion for big AMW DOM and continuous release, at least the low MW DOM, from POM in the deep sediments. These seemingly contrasting observations with the humification trends showed by optical and FT-ICR-MS data and abundant low MW probably arises from the fact that most of the low MW neutrals are both optically invisible and beyond the analytical windows of FT-ICR-MS probably due to preferential electrospray ionization.

This study is the first report on the CDOM and FDOM exponential production and insolation-paced oscillations in the deep sediment pore waters, and thus it fills the gap of organic matter biogeochemistry in deep oceanic sediments. The findings here can help to reconcile the bio-refractory and pre-aged nature of deep oceanic DOM since the sediments have been found to generally serve as sources of DOC and optical active DOM to the overlying water column^5, 34^. Furthermore, the insolation-paced paleoproductivity suggests potential higher organic carbon storage in tropical and subtropical sediments, assuming sufficient nutrients to support the primary productivity. Taken together, our study shed new light on close linkage of DOM quantity and composition and the carbon burial/preservation with paleoclimate such as the insolation, which were well reflected in deep marine sediments.

## Methods

### Sample collection

Pore waters and solid-phase sediments were chosen from all three sites and from non-chimney site U1, respectively. Pore water was extracted from all sediment samples (5–20 cm length) immediately after core retrieval. The surface of the sediments was scraped with a clean spatula or ceramic knife to eliminate any potential contamination with drilling fluid. The clean sediments were inserted into a titanium squeezer, which was modified from Manheim and Sayles^44^, and then extracted using a hydraulic press (<20 Mpa) to collect the pore water. The pore water was then filtered through a 0.20 µm pre-cleaned disposable polytetrafluoroethylene filter, and collected in acid-washed syringes. The sampled pore waters were contained in acid-washed Nalgene® high-density polyethylene bottles. The pore water and sediment samples for DOM analyses were immediately stored in a freezer. The samples for the onboard analyses and ion measurements were stored in a refrigerator (~4 °C).

The sediments from non-chimney site U1 were selected for alkaline extraction due to the high probability of the suitability for paleo-environmental reconstruction. The extraction and cleanup procedures of AEOM can be found elsewhere^45^. Briefly, dried sediments passed a 2 mm sieve, followed by soaking in 0.1 M NaOH solution and shaking for 24 h. Then the solution was centrifuged at 5000 rpm for 15 min. The supernatant were collected and filtered with a pre-washed 0.45 µm cellulose acetate filter (Advantec, Japan) and passed through cation exchange resin (DOWEX 50WX8-100, Sigma-Aldrich). An aliquot of the purified AEOM samples were diluted with ultrapure water prior to DOC, UV-Vis, and EEM measurements. The remaining elutes were concentrated with solid phase extraction (SPE) prior to FT-ICR-MS analysis.

### Analytical measurements

DOC concentrations were measured using a total organic carbon analyzer (Shimazu TOC-VCPH) as non-purgeable organic carbon with an analytical reproducibility of <2%^45^. The onboard measurements of pH, salinity, alkalinity, and chlorinity are described elsewhere^46^. Due to the volume limitation of pore waters, only 64 of 157 pore water samples were selected for optical measurements, and only 21 AEOM samples were further analyzed utilizing FT-ICR-MS. Absorption spectra were scanned from 240 to 800 nm with a Shimadzu 1800 ultraviolet-visible (UV-Vis) spectrophotometer (Shimadzu Inc., Japan). Fluorescence EEMs were scanned with a Hitachi F-7000 luminescence spectrometer (Hitachi Inc., Japan) at the excitation/emission wavelengths of 200–500/280–550 nm. The scanning speed was set at 12000 nm min^−1^. The excitation and emission slits were both 10 nm. The excitation was at 5 nm steps and emission at a 1 nm steps. All the samples were transferred into a capped cuvette in a nitrogen-filled glove box for the UV-Vis and EEMs measurements to minimize the redox potential changes.

SPE and FT-ICR-MS analyses were performed following the procedures described in previous studies^47, 48^ (The instrument is located in KBSI, Ochang, Korea). Detailed description is available in the supplementary information. SEC (chromatographic column: 250 × 20 mm, TSK, HW 50S; Toso, Japan) coupled with a UVD and an OCD (DOC Labor Dr. Huber, Karlsruhe, Germany) were performed to measure the MW distribution of PWDOM at non-chimney site U1. A mobile phase containing a phosphate buffer of pH 6.85 was used, and the flow rate was maintained at 1.1 mL/min. Detailed physical and method descriptions of this system can be found elsewhere^31, 32^. Due to the limited sample volume, six samples above the SMTZ were mixed at a 1:1 volume ratio for the analysis. Similarly, eight samples below the SMTZ and ≥118 mbsf were also mixed in a 1:1 volume ratio for SEC-UVD/OCD measurements. TOC and stable isotope δ^13^C measurements are available elsewhere^2^.

### Data processing

The UV-Vis absorption data were used to calculate the Napierian absorption coefficient at 254 and 350 nm, and to apply the inner filter effect correction^49, 50^. The absorption coefficient *a*_254_ is expected to reflect the overall DOM absorption, whereas *a*_350_ is usually related to the terrestrial-derived DOM such as lignin phenols^51, 52^. The correction procedures for post-acquisition of EEMs data are available elsewhere^30^. The corrected EEMs were then normalized by the integrated area of the Raman peak of daily measured Milli-Q water excited at 350 nm (Raman Unit: RU)^53^. PARAFAC modeling was performed using the MATLAB with the DOMFluor toolbox^54^. All the corrected PWDOM EEMs (n = 64) and AEOM EEMs (n = 21) were used separately for modeling to facilitate the comparison between them. The number of components was determined based on the split-half validation. The relative abundance of PARAFAC components, considered as chemical composition indices, was calculated with the percentage value of each component relative to the sum of the entire components in F_max_ intensity^55^. For FT-ICR-MS data, several selected indices such as intensity weighted average (wa) molecular masses, elemental ratios, element number, modified aromatic index (AI_mod_), and double bond equivalent (DBE) were calculated from the normalized peak intensities.

### First order kinetics model

A first order kinetic equation was found to fit the exponential production of bulk DOC, CDOM, FDOM, and nutrients and alkalinity as well as exponential reduction of sulfate in PWDOM. The equation is as follows:

1 $${{\rm{C}}}_{{\rm{t}}}={\rm{a}}\times {{\rm{e}}}^{({\rm{k}}\times {\rm{t}})}+{\rm{b}}$$

Where C_t_ is the total DOM, nutrients, alkalinity, or sulfate concentration at time t, a and b are constants, and k is the production (positive) or reduction (negative) rate. Time t was determined according to the age model in literature^1^ (Table S2). Likewise, the above Eq. 1 was also used to fit the exponential reduction of DOC, CDOM, and FDOM in AEOM.

## Electronic supplementary material

Supplementary Information
