# Supplementary material for: Biological early diagenesis and insolation-paced paleoproductivity signified in deep core sediment organic matter
Source: Sci Rep. 2017 May 8;7:1581. doi: 10.1038/s41598-017-01759-4 (PMC5431472; doi:10.1038/s41598-017-01759-4)
Supplement: Supplementary file 1 — Supplementary Information [file 41598_2017_1759_MOESM1_ESM.pdf]

Biological early diagenesis and insolation-paced paleoproductivity signified in deep core sediment organic matter

Meilian Chen[a], Ji-Hoon Kim[b], Jiyoung Choi[b], Yun Kyung Lee[a], and Jin Hur[a,*]

[a]*Department of Environment & Energy, Sejong University, Seoul 143-747, South Korea*

[b]*Petroleum and Marine Research Division, Korea Institute of Geoscience and Mineral Resources, 124 Gwahang-no, Yuseong-gu, Daejeon 34132, South Korea*

Re-submitted to *Scientific Reports*, February, 2017

* Corresponding author: E-mail: jinhur@sejong.ac.kr

Supporting Information: Table S1-S11 and Fig. S1-S14.

Site descriptions. The East Sea is a semi-closed marginal sea with a depth up to 3,500 m in the NW Pacific Ocean (Fig.1). It is connected to the modern open oceans with four shallow and narrow straits (~12, 55, 130, and 130 m). The Tsushima Warmer Current (a branch of the Kuroshio Current, ~1-2 Sv) carries relatively warm freshwater from the East China Sea, which receives discharge from the Yangtze River of China, into the East Sea through the Korea Strait (~130 m)[1]. The Tsugaru Strait between Honshu and Hokkaido (~130 m) is the main outlet of the East Sea. The turnover time of East Sea water by convection is about 100 years, and its residence time is approximately 1,000 years[2]. The Ulleung Basin, a deep, bowl-shaped, back-arc basin, is one of three basins (Japan, Yamato, and Ulleung) in the East Sea. This basin consists of four seismic units of turbidites/hemipelagics (Unit I), mass transport deposits (Units II and III), and a bottom unit (Unit IV)[3]. Seismic acoustic chimneys (i.e., vents/seeps) are common features in this basin, reaching up to ~2 km in width, and are concentrated in the center of the basin[4]. Most of these vertical-seismic terminate within the gas hydrate stability zone form truncated chimney sites, but a few could extend to the seafloor to form the seismic chimney sites[4,5].

During the Second Ulleung Basin Gas Hydrate Expedition (UBGH2) from July to September 2010, ten sites (< 250 mbsf or meters below seafloor) were drilled by *D/V Fugro Synergy*. Of those, three sites of UBGH2-1_1 (U1), UBGH2-2_1 (U2), and UBGH2-7 (U7), each representing a non-seismic chimney, a truncated seismic chimney (truncated ~76 mbsf), and a seismic chimney site, respectively, were selected for this study (Fig. 1 and Table S1). The water depths of these sites reached from ~1,500 to ~2,150 m. The uppermost unit of the sediments consists of turbidites and hemi-pelagic muds and/or mass-flow deposits.

Core sediments ~238 m deep generally correspond to the time up to ~2.5 Ma BP according to

the age-depth relations for this area, which coincides with the Pliocene-Pleistocene boundary[6,7]. The average sedimentation rates of late Quaternary sediments in this basin were 9 to 20 cm Kyr^{-1} based on tephra chronology and ^{14}C age dating[8,9]. The origins of the organic matter in the basin were reported to be dominantly marine algal source on the basis of the relationship between total organic carbon/total nitrogen (i.e., TOC/TN) and $\delta^{13}C_{org}$[10]. The location and characteristics of the sites are summarized in Table S1.

Water chemistry of deep oceanic sediment pore waters. The pore water chemistry (such as pH and ion strength) could potentially affect the optical behavior of DOM[11,12]. The pH measurements were 7.7 ± 0.3 ranging from 7.1 to 8.3 for all the samples (Table S1). The downcore profile of the pH was rather stable with the average values of 7.6 ± 0.2, 7.2 ± 0.3, and 7.7 ± 0.3, for sites U1, U2, and U7, respectively. The salinity showed relatively higher values at site U1 (student t-test, $p < 0.01$), probably due to the higher freshening effects (up to 53%) of upward migrating deep fluids, produced from clay dehydration/illitization, at the other two chimney sites of U2 and U7 than at site U1 (< 5%)[5]. For non-chimney site U1, the salinity was 36.6 ± 1.9. The bulk DOC data for U1, U2, and U7 pore waters showed the average concentrations of 8.9 ± 3.9, 4.4 ± 1.6, and 3.1 ± 1.2 mM, respectively.

Comparison of deep sediment DOM with those of water column. DOC and FDOM components of the PW-DOM in the Ulleung Basin(UB) were compared with those in other ecosystems including overlying seawater, and upstream pore waters (Table S3). As seen from the table, the

DOC spans a higher concentration range up to 21.2 mM for the system investigated here, orders of magnitude higher as compared to ~0.05 to 0.25 mM in the overlying water column[13,14], supporting the previous findings that the sediments generally serve as sources of DOC and CDOM to the overlying bottom waters[15,16]. It also indicates the efficient storage of dissolved organic carbon in the oceanic sediments. However, protein-like component, usually present in the overlying ocean, is missing in the deep oceanic sediments, suggesting that it may be generally relative labile and might have been decomposed during the long term diagenesis processes. The turnover of protein-like components has been reported to be on a timescale of hours, days, hundreds of years, and even thousands of years[17], depending on their sources, diagenetic status, and external conditions. Furthermore, all three humic-like components showed increasing trends with depth in the upper sediments, suggesting non-terrestrial source(s) for the traditionally termed terrestrial humic-like FDOM. Accumulation of humic-like FDOM in the mesopelagic and bathypelagic layers in water column and CDOM inputs from the deep sea to the euphotic zone have been reported in the East Sea[18,19]. Similar results were also observed in other oceanic studies[20,21].

The increasing trends of the three humic-like components in upper sediments also suggest that the accumulation of the humic-like FDOM could occur not only in the ocean interior[20], but also in upper sediments above SMTZ. The exclusive presence of humic-like components in deep sediments is consistent with the current understanding of the relative labile nature of protein DOM constituents, although protein-like FDOM components may be also associated with phenolic moieties besides proteinaceous materials[22]. In addition, the large deposit of DOM in deep sediments can be released into the overlying ocean through the vents and/or seeps as previously

reported[23]. Assuming these sites had a similar amount of original DOC levels and the sources, the amount released into overlying ocean could be quite significant since the DOC and CDOM values at the site U1 were about 2 to 3 times higher than those at the site U7 ((8.9 ± 3.9) vs. (3.1 ± 1.2) mM and (2.8 ± 0.9) vs. (1.3 ± 1.0) m^{-1}, respectively). Furthermore, as the DOM from deep sediments is pre-aged and relative refractory, the potential release from deep sediments may contribute to the estimated average age of DOC as well as the refractory nature of DOM in deep oceans.

Comparison between AEOM and PWDOM at site U1. The major differences are listed as follows: (1) The exponential increasing trend in PWDOM above SMTZ was opposite to that of AEOM, (2) Protein-like FDOM component was validated in AEOM (C3e) but not PW-DOM, probably due to "encapsulated" protein-like in particles which could be physically protected from degradation, (3) One order of magnitude higher FDOM signals were illustrated in AEOM than those in PWDOM, consistent with the previous reports that alkaline extraction can preferentially extract more humic-like DOM[24], (4) The **i**ncreasing HIX, humic-like components, and molecular weight trend with depth in AEOM suggested an intensified humification trend in the AEOM, which was not observed in naturally occurring PWDOM. (5) AEOM seemed less affected by deep fluids migration than PWDOM, consistent with its solid phase nature.

Classification of sub-groups at site U1 via cluster analysis based on AEOM DOC, optical, and molecular data. Cluster Analysis (Ward Method) was performed based on the DOC, optical,

molecular, and elemental data of AEOM (Fig. S7). Three sub-groups were classified primarily based on the depth, except for one outlier at depth of 179 mbsf (~1.3-Ma). The sub-group #1 (G1) coincided with the SMTZ depth at ~21 mbsf and the sub-group #3 (G3, > 118 mbsf) was assumed to be the depth significantly affected by basal fluids upward migration. As stated before, the sub-group #2 (G2) was characterized by insolation-paced oscillations since it was affected by neither the active microbial activity nor the geological effects. The characteristics of optical features of each sub-group as well as unique and common molecular formula are illustrated in Fig. S7-S8 and Table S6-S7, S9. As seen from the results, G1, G2, and G3 exhibited a broad spectrum of molecular formula, which fall in the range of lipids, proteins, carbohydrates, lignin-like/CRAM (i.e., carboxyl-rich acyclic molecules), unsaturated hydrocarbons, and condensed aromatic structures in van Krevelen diagrams. Principal component analysis (PCA) showed that G1 was primarily affected by protein-like component and H and O elements, while G2 and G3 were mainly controlled by humic-like substances and molecular weight (Fig. S12).

Humification with depth for AEOM. Humification of AEOM (e.g., increases of aromaticity proxy $a(254)^*$, m/z_{wa}, humic-like FDOM, and humification index) was observed with depth (Fig. S4 and Fig. S6). The definitions of humification index (HIX) and biological index (BIX) can be found elsewhere[25,26]. The linear increase of absolute abundance of C1e, C2e, and C3e were obvious with the R^2 values ranging from 0.4-0.8 ($p < 0.01$). For the relative abundance (i.e., composition), humic-like components, C1e and C3e, showed increasing trends ($R^2 = 0.4$ and 0.6, respectively, $p < 0.05$ and $p < 0.0001$) as opposite to that of protein-like C3e ($R^2 = 0.6$, $p < 0.0001$). Meanwhile,

HIX displayed an increasing trend with depth ($R^2 = 0.6$, $p < 0.0001$) as opposite to that of BIX ($R^2 = 0.4$, $p < 0.01$). Aromatic proxy of *a*(254)* also showed a linear increasing trend with a R^2 value of 0.2. Similarly, molecular data also featured increasing trends. For example, linear increasing trends were found for m/z_{wa} ($R^2 = 0.4$; $p < 0.05$) and S_{wa} ($R^2 = 0.3$; $p < 0.05$). Chemodiversity of molecular formula number (15,281), abundant heteroatomic elements of S and N, and increasing S_{wa} with depth are interesting findings. Early diagenetic sulfurization of organic matter were reported previously[15,27], which were suggested to occur primarily via abiotic incorporation of organic matter with H_2S or polysulphides[28].

SPE and FT-ICR-MS procedures. In brief, the cartridges were rinsed with methanol and washed extensively with acidified ultrapure water prior to use. One liter of the acidified sample was discharged through the cartridge, dried, and then eluted with methanol. The samples were immediately stored in freezer, which after FT-ICR-MS measurements were done within 1 week. The analysis was carried out with a 15-T FT-ICR-MS interfaced with an Appollo II electrospray ionization source (ESI, Bruker Daltonik, Germany). The instrument was run in broadband mode between a 160–1000 m/z range in negative ion mode. The samples were injected at a flow rate of 2 µl/min. Negatively charged ions were accumulated in an argon-filled collision cell for 1 s. One hundred transient scans were co-added to one mass spectrum. External calibration with arginine clusters and internal recalibration with SRFA200-900 were made to achieve a mass accuracy of < 0.03 ppm. An average resolving power ($m/\Delta m_{50\%}$) of > 300,000 at m/z ~400 was routinely achieved. Procedural blanks were run and subtracted from each corresponding sample. Solvent blanks were run before and after each sample. A magnitude threshold was set to an S/N ratio of ≥

4. All ions were singly charged as confirmed by the isotopic spacing pattern (1.00335 Da) of the corresponding $^{12}C_n$ and $^{13}C^{12}C_{n-1}$ mass peaks. Molecular formulas were calculated allowing for the elemental combinations of $^{12}C_{0\text{-}\infty}{}^{1}H_{0\text{-}\infty}{}^{16}O_{0\text{-}\infty}{}^{14}N_{0\text{-}4}{}^{32}S_{0\text{–}2}$. The mass accuracy threshold was $|\Delta m| \leq 1$ ppm. The following elemental ratio criteria were implemented: 2.25 > H/C > 0.3, O/C < 1.2, N/C < 0.5, S/C < 0.2.

Downcore profile of DOC-normalized CDOM and FDOM parameters. As seen from Fig. S14, the DOC-normalized CDOM and FDOM parameters generally remained constant after the initial decrease to the depth of SMTZ, consistent with faster production rate of DOC relative to CDOM and FDOM parameters above SMTZ (0.05 Kyr^{-1} for DOC versus 0.01-0.03 Kyr^{-1} for CDOM and FDOM parameters, Table 1). The profile also agrees well with the general trend of correlation between DOC and the other parameters of PWDOM (correlation coefficients: 0.4-0.8, Table S11).

References:

1. Isobe, A. *et al.* Freshwater and temperature transports through the Tsushima-Korea Straits. *Journal of Geophysical Research: Oceans* **107**, 2-1-2-20 (2002).

2. Watanabe, Y. W., Watanabe, S. & Tsunogai, S. Tritium in the Japan Sea and the renewal time of the Japan Sea deep water. *Mar. Chem.* **34**, 97-108 (1991).

3. Chough, S. K., Lee, H. J. & Yoon, S. H. *Marine geology of Korean seas*. Vol. 2nd Eds. (Elsevier, 2000).

4. Horozal, S. *et al.* Late Neogene–Quaternary submarine mass wasting along the margins of the Ulleung Basin, East Sea: Geomorphologic controls and geohazard potential. *Quaternary International* **392**, 69-98 (2016).

5. Kim, J.-H. *et al.* Pore fluid chemistry from the Second Gas Hydrate Drilling Expedition in the Ulleung Basin (UBGH2): Source, mechanisms and consequences of fluid freshening in the central part of the Ulleung Basin, East Sea. *Marine and Petroleum Geology* **47**, 99-112 (2013).

6. Bahk, J.-J., Um, I.-K., Yi, B.-Y. & Yoo, D.-G. Paleoceanographic implications and cyclostratigraphy of variations in well-log data from the western slope of the Ulleung Basin, East Sea. *Quaternary International* **392**, 58-68 (2016).

7. Yi, S., Bahk, J.-J., Jia, H. & Yoo, D.-G. Pliocene–Pleistocene boundary determination in hemipelagic sediment from the Ulleung Basin (East Sea, offshore Korea) inferred from terrigenous and marine palynofloras. *Review of Palaeobotany and Palynology* **181**, 54-63 (2012).

8. Park, M. H., Kim, J. H., Ryu, B. J., Kim, I. S. & Chang, H. W. AMS radiocarbon dating of the marine late Pleistocene-Holocene sediment cores from the Ulleung Basin, East/Japan Sea. *Nucl. Instr. Meth. Phys. Res. B* **243**, 211-215 (2006).

9. Park, M. H., Kim, J. H. & Kil, Y. W. Identification of the late Quaternary tephra layers in the Ulleung Basin of the East Sea (Sea of Japan) using geochemical and statistical methods. *Mar. Geol.* **244**, 196-208 (2007).

10. Kim, J.-H., Kong, G.-S., Ryu, J.-S. & Park, M.-H. Revisiting the origin of organic matter and depositional environment of sediment in the central Ulleung Basin, East Sea since the late Quaternary. *Quaternary International* **344**, 181-191 (2014).

11. Zepp, R. G., Sheldon, W. M. & Moran, M. A. Dissolved organic fluorophores in southeastern US coastal waters: correction method for eliminating Rayleigh and Raman scattering peaks in excitation–emission matrices. *Mar. Chem.* **89**, 15-36 (2004).

12. Pace, M. *et al.* pH change induces shifts in the size and light absorption of dissolved organic matter. *Biogeochem.* **108**, 109-118 (2012).

13. Yamaguchi, Y., Nakaguchi, Y., Hattori, H., Kimura, M. & Hiraki, K. Anomalous distribution of dissolved organic carbon in the Sea of Japan. *Geochemical Journal* **35**, 355-364 (2001).

14. Kim, T. H., Kim, G., Lee, S. A. & Dittmar, T. Extraordinary slow degradation of dissolved organic carbon (DOC) in a cold marginal sea. *Scientific Reports* **5** (2015).

15. Burdige, D. J. & Komada, T. *Sediment pore waters*. 2nd ed., 536-569 (Academic Press, 2014).

16. Chen, M. & Hur, J. Pre-treatments, characteristics, and biogeochemical dynamics of dissolved organic matter in sediments: A review. *Water Research* **79**, 10-25, (2015).

17. Chen, M. & Jaffé, R. Quantitative assessment of photo- and bio-reactivity of chromophoric and fluorescent dissolved organic matter from biomass and soil leachates and from surface waters in a subtropical wetland. *Biogeochemistry* doi:10.1007/s10533-016-0231-7 (2016).

18. Tanaka, K., Kuma, K., Hamasaki, K. & Yamashita, Y. Accumulation of humic-like fluorescent dissolved organic matter in the Japan Sea. *Scientific Reports* **4**, doi:529210.1038/srep05292 (2014).

19. Kim, J. & Kim, G. Importance of colored dissolved organic matter (CDOM) inputs from the deep sea to the euphotic zone: Results from the East (Japan) Sea. *Marine Chemistry* **169**, 33-40 (2015).

20. Yamashita, Y. & Tanoue, E. Production of bio-refractory fluorescent dissolved organic matter in the ocean interior. *Nature Geosci* **1**, 579-582 (2008).

21. Jørgensen, L. *et al.* Global trends in the fluorescence characteristics and distribution of marine dissolved organic matter. *Marine Chemistry* **126**, 139-148 (2011).

22. Maie, N., Scully, N. M., Pisani, O. & Jaffé, R. Composition of a protein-like fluorophore of dissolved organic matter in coastal wetland and estuarine ecosystems. *Water Research* **41**, 563-570 (2007).

23. Pohlman, J. W., Bauer, J. E., Waite, W. F., Osburn, C. L. & Chapman, N. R. Methane hydrate-bearing seeps as a source of aged dissolved organic carbon to the oceans. *Nature Geosci* **4**, 37-41, doi:http://www.nature.com/ngeo/journal/v4/n1/abs/ngeo1016.html#supplementary-information (2011).

24. Hur, J., Lee, B. M. & Shin, K. H. Spectroscopic characterization of dissolved organic matter isolates from sediments and the association with phenanthrene binding affinity. *Chemosphere* **111**, 450-457 (2014).

25. Zsolnay, A., Baigar, E., Jimenez, M., Steinweg, B. & Saccomandi, F. Differentiating with fluorescence spectroscopy the sources of dissolved organic matter in soils subjected to drying. *Chemosphere* **38**, 45-50 (1999).

26. Huguet, A. *et al.* Properties of fluorescent dissolved organic matter in the Gironde Estuary. *Organic Geochemistry* **40**, 706-719 (2009).

27. Schmidt, F., Elvert, M., Koch, B. P., Witt, M. & Hinrichs, K.-U. Molecular characterization of dissolved organic matter in pore water of continental shelf sediments. *Geochimica Et Cosmochimica Acta* **73**, 3337-3358 (2009).

28. Sinninghe Damste, J. S. & De Leeuw, J. W. Proceedings of the 14th International Meeting on Organic Geochemistry Analysis, structure and geochemical significance of organically-bound sulphur in the geosphere: State of the art and future research. *Organic Geochemistry* **16**, 1077-1101 (1990).

29. Wang, Y., Zhang, D., Shen, Z. Y., Chen, J. & Feng, C. H. Characterization and spacial distribution variability of chromophoric dissolved organic matter (CDOM) in the Yangtze Estuary. *Chemosphere* **95**, 353-362 (2014).

30. Wang, Y., Zhang, D., Shen, Z. Y., Feng, C. H. & Chen, J. Revealing Sources and Distribution Changes of Dissolved Organic Matter (DOM) in Pore Water of Sediment from the Yangtze Estuary. *Plos One* **8** (2013).

31. Coble, P. G. Characterization of marine and terrestrial DOM in seawater using excitation-emission matrix spectroscopy. *Marine Chemistry* **51**, 325-346 (1996).

32. Berger, A. in *IGBP PAGES/World Data Center for Paleoclimatology* Vol. Data Contribution Series #92-007, ftp://ftp.ncdc.noaa.gov/pub/data/paleo/insolation/ (NOAA/NGDC Paleoclimatology Program, Boulder CO, USA, 1992) (Date of access: 19/10/2016).

33. Ryu, B.-J. *et al.* The Second Ulleung Basin Gas Hydrate Drilling Expedition (UBGH2) Expedition Report. (Korea Institute of Geoscience and Mineral Resources (KIGAM), Daejeon, Republic of Korea, 2012).

34. Hong, W.-L., Torres, M., Kim, J.-H., Choi, J. & Bahk, J.-J. Carbon cycling within the sulfate-methane-transition-zone in marine sediments from the Ulleung Basin. *Biogeochemistry* **115**, 129-148 (2013).

35. Kim, J.-H. *et al.* Pore fluid chemistry from the Second Gas Hydrate Drilling Expedition in the Ulleung Basin (UBGH2): Source, mechanisms and consequences of fluid freshening in the central part of the Ulleung Basin, East Sea. *Marine and Petroleum Geology* **47**, 99-112 (2013).

36. Choi, J. *et al.* Gas origin and migration in the Ulleung Basin, East Sea: Results from the Second Ulleung Basin Gas Hydrate Drilling Expedition (UBGH2). *Marine and Petroleum Geology* **47**, 113-124 (2013).

Table S1. Summary of site loci, water chemistry, and DOC and optical characteristics (mean ± SD).

Site	UBGH2-1_1 (U1)		UBGH2-2_1 (U2)	UBGH2-7 (U7)
Latitude (N)	36º 15' 4.8"		36º 42' 43.1"	36º 54' 53.7"
Longitude (E)	130º 3' 56.6"		130º 52' 56.4"	130º 22' 00.6"
Water depth (m)	1,529.30		2,092.20	2,144.60
Total drilled depth (mbsf)	216		192	238
Seismic characteristics	Non-chimney		Truncated chimney	Chimney
pH	7.6 ± 0.2		7.7 ± 0.3	7.7 ± 0.3
Salinity	36.6 ± 1.9		27.2 ± 5.3	31.1 ± 9.4
	PWDOM	AEOM	PWDOM	PWDOM
DOC (mM)	8.9 ± 3.9	2.1 ± 1.0	4.4 ± 1.6	3.1 ± 1.2
a_{254} (m^{-1})	10.0 ± 4.3	9.1 ± 4.7	3.5 ± 1.7	3.2 ± 2.7
a_{350} (m^{-1})	1.2 ± 0.4	2.5 ± 1.3	0.6 ± 0.3	0.6 ± 0.4
a_{254}* ($L(mgC\text{-}m)^{-1}$)	0.1 ± 0.0	0.4 ± 0.1	0.1 ± 0.0	0.1 ± 0.1
BIX	0.8 ± 0.0	0.8 ± 0.1	0.8 ± 0.0	0.8 ± 0.0
HIX	9.5 ± 0.2	6.4 ± 2.8	9.7 ± 0.1	9.7 ± 0.1
C1[ƚ]	0.19 ± 0.0	2.9 ± 1.6	0.19 ± 0.0	0.19 ± 0.0
C2[ƚ]	0.19 ± 0.0	1.5 ± 0.8	0.19 ± 0.0	0.19 ± 0.0
C3[ƚ]	0.20 ± 0.0	2.7 ± 0.3	0.20 ± 0.0	0.20 ± 0.0
C1%[ƚ]	32.8 ± 0.1	37.2 ± 10.6	32.7 ± 0.1	32.6 ± 0.1
C2%[ƚ]	32.6 ± 0.3	20.6 ± 4.3	33.0 ± 0.2	33.1 ± 0.3
C3%[ƚ]	34.6 ± 0.2	42.2 ± 14.0	34.3 ± 0.1	34.3 ± 0.2
SO_4^{2-} (mM)	6.5 ± 9.2	–	6.2 ± 9.1	4.2 ± 10.4
NH_4^+ (mM)	10.6 ± 5.7	–	70.8 ± 57.5	109.2 ± 109.7

PO_4^{3-} (μM)	229.8 ± 136.3	–	0.9 ± 0.1	0.8 ± 0.4
Alkalinity (mM)	91.4 ± 44.1	–	33.5 ± 12.5	33.6 ± 20.6
Chlorinity (mM)	537.8 ± 5.0	–	433.0 ± 90.5	504.9 ± 151.0

[ł]Note that EEM-PARAFAC components (C1-C3) are different between PWDOM and AEOM.

Table S2. Age-depth relation for the site U1 (data derived from literature)[6].

Depth (mbsf)	Age (Ka)
0.2	0.4
6.1	11.1
9.6	40.8
12.1	69.2
17.8	103.3
19.6	119.9
24.0	132.1
41.2	215.6
41.8	223.2
44.0	239.6
49.5	274.5
54.0	309.4
57.5	335.0
65.0	387.0
69.3	405.3
75.3	433.8
82.0	498.0
104.6	621.5
109.0	649.3
111.6	670.5
116.2	701.8
119.2	725.5
121.5	752.4
125.6	782.2
127.1	795.6
133.4	836.3
137.4	872.5
140.2	910.1
142.1	927.2
145.0	966.8
151.4	1042.7
155.9	1107.7
159.8	1143.0
162.3	1161.7
177.5	1281.5
183.9	1345.8
186.6	1389.4

(Continued)

Depth (mbsf)	Age (Ka)
195.0	1465.3
203.0	1513.4
206.9	1551.5
214.2	1598.9
216.5	1613.0
220.8	1635.0
227.5	1664.0
230.6	1691.9

Table S3. Comparison of the DOC and EEM-PARAFAC components in this study with other related marine systems.

Ecosystem			Findings				Reference
	Depth (m)	DOC (mM)	component[ǂ]	Ex/Em maxima (nm)	assignment (traditional*)	abundance (%)	
PWDOM	0-236.5	0.5-21.2	C1p	295/395	marine humic-like (M)	~32-33	In this study
			C2p	340/430	humic-like (C)	~32-33	
			C3p	<260/470	humic-like (A)	~34-35	
AEOM	218.1	0.8-4.6	C1e	<260(315)/420	marine humic-like (M)	19-51	In this study
			C2e	(260)390/478	humic-like (A, C)	13-26	
			C3e	295/305	protein-like	23-68	
Water column (Overlying)	0-4,500	-	C1	<260(350)/470	terrestrial humic-like (A,C)	-	Ref. 18
			C2	<260(320)/395	marine humic-like (M)	-	
			C3	<260/<290	protein-like	-	
	0-4,000	-	C1	325/404	marine humic-like (M)	-	Ref. 19
			C2	285/336	protein-like (T)	-	
			C3	270(395)/452	terrestrial humic-like (A,C)	-	
	0-4,000	~0.06-0.09	-	-	-		Ref. 14
	0-4,000	~0.05-0.25	-	-	-	-	Ref. 13
Coastal porewater Yangtze Estuary (Upstream)	surface	6.1-6.8	C1	<260/425	terrestrial humic-like (A,C)	-	Ref. 29
			C2	<240(280)/340	protein-like (T)	-	
			C3	<260(360)/475	terrestrial humic-like (A,C)	-	

Yangtze Estuary (Upstream)	0.25	1.7-37.3	C1	<240(300)/410	marine humic-like (M)	~20-30	Ref. 30
			C2	265(365)/470	terrestrial humic-like (C,A)	~10-20	
			C3	<240(275)/335	protein-like (T)	~20-35	

[ǂ]Note same component C1-C3 are different for different datasets. *Based on Coble[31].

Table S4. Net production (+) and reduction (-) and kinetics rates (unit: Kyr^{-1}) of AEOM and water chemistry parameters at site U1.

AEOM:	DOC	a_{254}	a_{350}	C1e‡	C2e‡	C3e‡	HIX	a_{254}*	m/z_{wa}	C_{wa}	S_{wa}	N_{wa}	S/C_{wa}
unit	mM	m^{-1}	m^{-1}	RU	RU	RU		$L(mgC\text{-}m)^{-1}$	Da				
Net reduction‡	-1.7	-4.0	-2.3	-0.3	-0.6	-0.1	–	0.2	17	0.7	0.2	-0.3	0.008
$k_{SMTZ,1st\ order}$	-6.9	-7.1	-6.8	–	-7.4	–	–	–	–	–	–	–	–
$k_{all,linear}$	0.0007	0.005	0.0006	0.003	0.001	0.0003	0.004	0.00007	0.02	0.001	0.0001	–	0.000006

‡The value at SMTZ depth (~21 mbsf) minus that at the depth of zero.

Nonsignificant regressions are not shown.

Table S5. Matrix of linear correlation coefficients of DOM, ions, and nutrients parameters above SMTZ at site U1 ($p < 0.01$).

Item:	DOC	a_{254}	a_{350}	C1p	C2p	C3p	SO_4^{2-}	NH_4^+	PO_4^{3-}	Alkalinity
DOC										
a_{254}	0.94									
a_{350}		0.93								
C1p	0.89	0.98	0.98							
C2p										
C3p	0.90	0.99	0.97	1.00						
SO_4^{2-}	-0.95	-0.93		-0.90		-0.90				
NH_4^+	0.97	0.97		0.92		0.93	-0.95			
PO_4^{3-}	0.94	0.97		0.90		0.92	-0.93	0.99		
Alkalinity	0.97	0.97		0.92		0.93	-0.98	0.99	0.98	

Table S6. Non-redundant, common and unique molecular characteristics of AEOM among different depth.

Item	Non-redundant	Common	Unique					
		G1, 2, 3	G1, 2	G1, 3	G2, 3	G1	G2	G3
# Formula	15281	4578	913	1600	1602	2314	1618	2413
m/z_{wa}	405	369	402	430	438	440	407	464
$AI_{mod, wa}$	0.40	0.40	0.45	0.21	0.64	0.30	0.60	0.51
DBE_{wa}	12.6	11.7	13.3	9.3	18.5	11.6	15.6	17.4
C_{wa}	22.3	20.1	22.6	21.0	26.3	24.1	23.6	26.8
H_{wa}	22.3	19.7	21.8	25.8	18.7	28.0	19.4	21.9
O_{wa}	5.2	5.1	4.7	7.4	3.9	5.7	3.8	4.9
N_{wa}	0.90	0.83	1.17	0.41	1.02	0.95	1.40	1.08
S_{wa}	0.62	0.44	0.55	0.83	0.82	0.57	0.73	0.84
H/C_{wa}	1.07	1.05	1.02	1.27	0.74	1.23	0.85	0.91
O/C_{wa}	0.28	0.31	0.25	0.40	0.18	0.28	0.19	0.25
N/C_{wa}	0.04	0.04	0.06	0.02	0.05	0.04	0.07	0.05
S/C_{wa}	0.03	0.03	0.03	0.04	0.03	0.03	0.03	0.04
# CHO-only	2036(13%)	824(18%)	81(9%)	170(11%)	151(9%)	428(18%)	115(7%)	239(10%)
# CHOS	8824(58%)	2104(46%)	495(54%)	1206(75%)	1170(73%)	1132(49%)	947(60%)	1618(67%)
# CHON	9974(65%)	2961(65%)	693(76%)	628(39%)	1176(73%)	1442(62%)	1283(79%)	1588(66%)
# CHONS	5553(36%)	1311(29%)	356(39%)	405(25%)	894(56%)	688(30%)	740(46%)	1032(43%)

G1: depth ≤ 21 m; G2: 21 < depth ≤ 118 m; G3: depth > 118 m.

Table S7. Molecular characteristics of AEOM among three groups (G1-3).

Molecular:	G1<br>depth ≤ 21 m	G2<br>21 < depth ≤ 118 m	G3<br>depth > 118 m
# Formula	3497 ± 821(9423)	3326 ± 497(8720)	3867 ± 332(10216)
m/z_{wa}	360 ± 19	367 ± 10	383 ± 12
$AI_{mod, wa}$	0.4 ± 0.1	0.5 ± 0.0	0.4 ± 0.1
DBE_{wa}	9.9 ± 0.9	12.7 ± 0.5	12.3 ± 0.7
C_{wa}	19.1 ± 1.0	20.4 ± 0.6	20.9 ± 0.7
H_{wa}	21.2 ± 2.2	18.4 ± 0.4	19.9 ± 1.3
O_{wa}	5.3 ± 0.3	4.7 ± 0.2	5.1 ± 0.3
N_{wa}	0.80 ± 0.14	1.04 ± 0.07	0.83 ± 0.13
S_{wa}	0.40 ± 0.12	0.39 ± 0.08	0.57 ± 0.12
H/C_{wa}	1.16 ± 0.08	0.95 ± 0.03	0.98 ± 0.06
O/C_{wa}	0.33 ± 0.02	0.28 ± 0.01	0.30 ± 0.02
N/C_{wa}	0.04 ± 0.01	0.05 ± 0.00	0.04 ± 0.01
S/C_{wa}	0.02 ± 0.01	0.02 ± 0.00	0.03 ± 0.01

G1: depth ≤ 21 m; G2: 21 < depth ≤ 118 m; G3: depth > 118 m.

Table S8. Linear correlation coefficients (expressed as R^2) between insolation (15°N in July) and AEOM parameters at non-chimney site U1.

AEOM:	DOC mM-C	a_{254} m^{-1}	C1e RU	C2e RU	C3e RU	m/z_{wa} Da	HIX	C_{wa}	S_{wa}
R^2	0.3	0.3	0.3	0.2	–	0.2	0.3	0.1	0.1
Amplitude*	2.3	2.1	2.3	1.7	0.7	18.1	1.7	0.8	0.1
ΔDOM/Δinsolation[ǂ]	0.06	0.04	0.06	0.05	0.02	0.50	0.05	0.02	0.004

Correlation excluding the depth above SMTZ (21 mbsf) and below 118 mbsf (affected by fluids migration) ($p < 0.05$).

*[ǂ]Estimated using an insolation rising phase from depth 68 to 118 mbsf (399- to 718-Ka). Insolation unit : Wm^{-2}.

Table S9. Characteristics of DOC and optical features of three sub-groups (G1, G2, and G3) of AEOM.

Item	Unit	G1<br>depth ≤ 21 m	G2<br>21 < depth ≤ 118 m	G3<br>depth > 118 m
DOC	mM	1.4 ± 0.7	2.1 ± 0.6	3.0 ± 0.9
a_{254}	m^{-1}	5.4 ± 1.9	8.0 ± 1.8	14.9 ± 4.3
a_{350}	m^{-1}	2.3 ± 0.9	2.0 ± 0.9	3.7 ± 1.6
a_{254}*	$L(mgC\text{-}m)^{-1}$	0.3 ± 0.1	0.3 ± 0.1	0.4 ± 0.1
BIX		0.9 ± 0.1	0.8 ± 0.1	0.7 ± 0.0
HIX		3.3 ± 0.4	6.4 ± 1.7	9.9 ± 1.2
C1	RU	1.0 ± 0.2	2.7 ± 0.7	4.9 ± 0.5
C2	RU	0.7 ± 0.3	1.6 ± 0.6	2.3 ± 0.3
C3	RU	2.4 ± 0.2	2.7 ± 0.4	2.7 ± 0.2
C1	%	23.5 ± 2.8	38.3 ± 5.7	49.1 ± 1.4
C2	%	16.7 ± 3.9	21.7 ± 4.0	23.6 ± 1.4
C3	%	59.7 ± 6.1	40.0 ± 8.0	27.3 ± 2.5
Chlorinity	mM	541 ± 7	538 ± 3	536 ± 3

Table S10. Total organic carbon (TOC) and organic carbon stable isotope data for sediment cores.

U1			U2			U7		
Depth (mbsf)	TOC (wt.%)	$\delta^{13}C$ (‰)	Depth (mbsf)	TOC (wt.%)	$\delta^{13}C$ (‰)	Depth (mbsf)	TOC (wt.%)	$\delta^{13}C$ (‰)
0.3	1.8	-21.9	0.3	n/a	n/a	0.00	n/a	n/a
0.6	1.7	-22.3	0.7	1.8	-22.9	0.04	n/a	n/a
1.0	1.7	n/a	1.5	3.0	-23.1	0.3	n/a	n/a
1.3	1.8	-22.4	2.2	1.2	-23.5	3.0	3.6	-24.4
1.7	1.8	n/a	3.0	1.5	-23.5	3.7	1.1	n/a
2.0	1.8	-22.5	3.7	1.6	n/a	4.5	1.3	-23.4
2.4	1.8	n/a	6.0	0.7	-24.4	11.1	1.3	n/a
2.7	2.0	-22.4	6.7	1.4	-24.2	12.45	1.8	-23.4
3.1	2.0	n/a	9.0	3.7	-23.5	12.60	2.2	-23.8
3.4	2.1	-22.5	12.0	1.5	-23.5	14.1	n/a	n/a
3.5	2.1	n/a	13.5	1.3	n/a	25.0	1.4	-24.3
4.1	2.2	-22.5	30.4	1.3	n/a	25.1	1.3	-24.1
4.5	2.4	n/a	46.6	1.0	-26.2	27.0	1.8	n/a
5.5	4.0	-24.1	63.9	0.8	-23.8	84.6	2.3	-23.5
5.9	1.1	n/a	77.7	2.2	-23.7	122.2	1.4	-24.9
6.6	1.6	-24.7	97.6	1.1	-22.8	172.4	1.1	-22.8
7.2	1.8	n/a	104.5	2.0	-22.3	198.7	0.9	-23.5
3.5	2.2	n/a	111.2	2.2	-21.8	231.4	0.9	-22.8
7.5	1.1	n/a	122.6	1.7	-22.0	236.5	0.6	-23.1
8.5	1.3	-25.0	137.4	1.5	-21.5			
9.0	0.8	n/a	149.2	1.5	-21.6			
9.4	0.8	-23.9	170.5	1.6	-21.9			

13.0	2.7	n/a	180.5	1.8	-21.9
16.0	2.0	-22.8			
17.5	2.2	-23.2			
21.0	1.4	-23.2			
24.0	1.0	-25.2			
27.5	3.1	n/a			
31.9	2.8	-22.4			
37.0	2.8	n/a			
40.0	2.5	-22.7			
44.6	1.0	-25.0			
47.7	3.2	-22.8			
52.7	1.6	-23.0			
55.9	2.7	n/a			
60.7	1.6	-23.6			
63.7	2.0	n/a			
67.7	1.5	-23.0			
70.7	1.0	n/a			
75.4	1.9	-23.3			
78.4	2.3	n/a			
84.5	2.8	-22.2			
87.0	3.1	-23.1			
93.3	2.3	n/a			
93.6	0.1	n/a			
93.7	4.0	-23.7			
93.8	2.1	-23.3			
96.1	2.2	n/a			
100.3	1.5	-24.1			
101.0	2.8	n/a			

101.1	1.8	-23.5
103.6	2.2	n/a
108.4	1.9	n/a
111.2	2.9	-24.3
115.2	2.4	-23.1
118.2	1.5	-24.5
119.1	1.7	-23.6
126.4	1.1	-26.3
128.4	0.7	n/a
130.9	1.3	-23.2
133.0	1.7	n/a
133.7	2.5	-23.4
133.8	2.7	n/a
134.0	2.7	n/a
134.9	1.5	-23.1
142.1	3.2	-22.2
144.9	3.0	n/a
149.7	2.3	n/a
152.1	2.3	-22.7
155.8	2.7	n/a
155.9	2.6	n/a
158.5	3.0	-22.9
161.0	1.8	-22.6
168.1	1.4	-24.8
171.1	1.3	n/a
175.9	0.8	n/a
178.9	0.8	-23.1
186.4	0.6	n/a

189.2	1.1	-22.6
194.4	1.2	n/a
198.3	1.3	-22.0
207.0	1.7	n/a
209.7	1.3	-23.2
215.1	1.3	n/a
218.1	1.6	-22.0

n/a: not measured.

Table S11. Matrix of linear correlation coefficients among insolation at 15 N° in July and DOM parameters.

		PWDOM:						AEOM:												
	Insolation	DOC	a_{254}	a_{350}	C1p	C2p	C3p	DOC	a_{254}	a_{350}	C1e	C2e	C3e	m/z_{wa}	C_{wa}	N_{wa}	S_{wa}	N/C_{wa}	S/C_{wa}	TOC
Insolation																				
PWDOM:																				
DOC	0.5																			
a_{254}	0.6	0.4																		
a_{350}	0.4	0.8	0.6																	
C1p	0.6	0.6	0.9	0.9																
C2p	0.4	0.8	0.4	1.0	0.8															
C3p	0.6	0.4	1.0	0.8	1.0	0.6														
AEOM:																				
DOC	0.5	0.6	0.3	0.4	0.4	0.4	0.3													
a_{254}	0.5		0.5	0.2	0.4		0.5	0.3												
a_{350}	0.3		0.6		0.4		0.5		0.7											
C1e	0.5	0.6		0.4	0.3	0.4		0.8	0.4											
C2e	0.4	0.8	0.3	0.6	0.5	0.6	0.4	0.9			0.8									
C3e		0.7	0.4	0.8	0.6	0.8	0.4	0.7			0.4	0.8								
m/z_{wa}	0.4	0.6		0.6	0.4	0.7	0.3	0.7			0.5	0.8	0.8							
C_{wa}	0.3	0.6		0.5	0.3	0.7		0.6			0.4	0.7	0.8	1.0						
N_{wa}									0.3	0.3			-0.6							
S_{wa}	0.4	0.4	0.6	0.5	0.6	0.4	0.7	0.6	0.4		0.4	0.6	0.5	0.6	0.4					
N/C_{wa}	0.4								0.4	0.5			-0.6			0.9				
S/C_{wa}	0.4	0.4	0.6	0.5	0.7	0.4	0.7	0.6	0.4			0.5	0.5	0.5	0.4		1.0			
TOC	0.3	0.3	0.4				0.2	0.3	0.6	0.8						0.7		0.8		

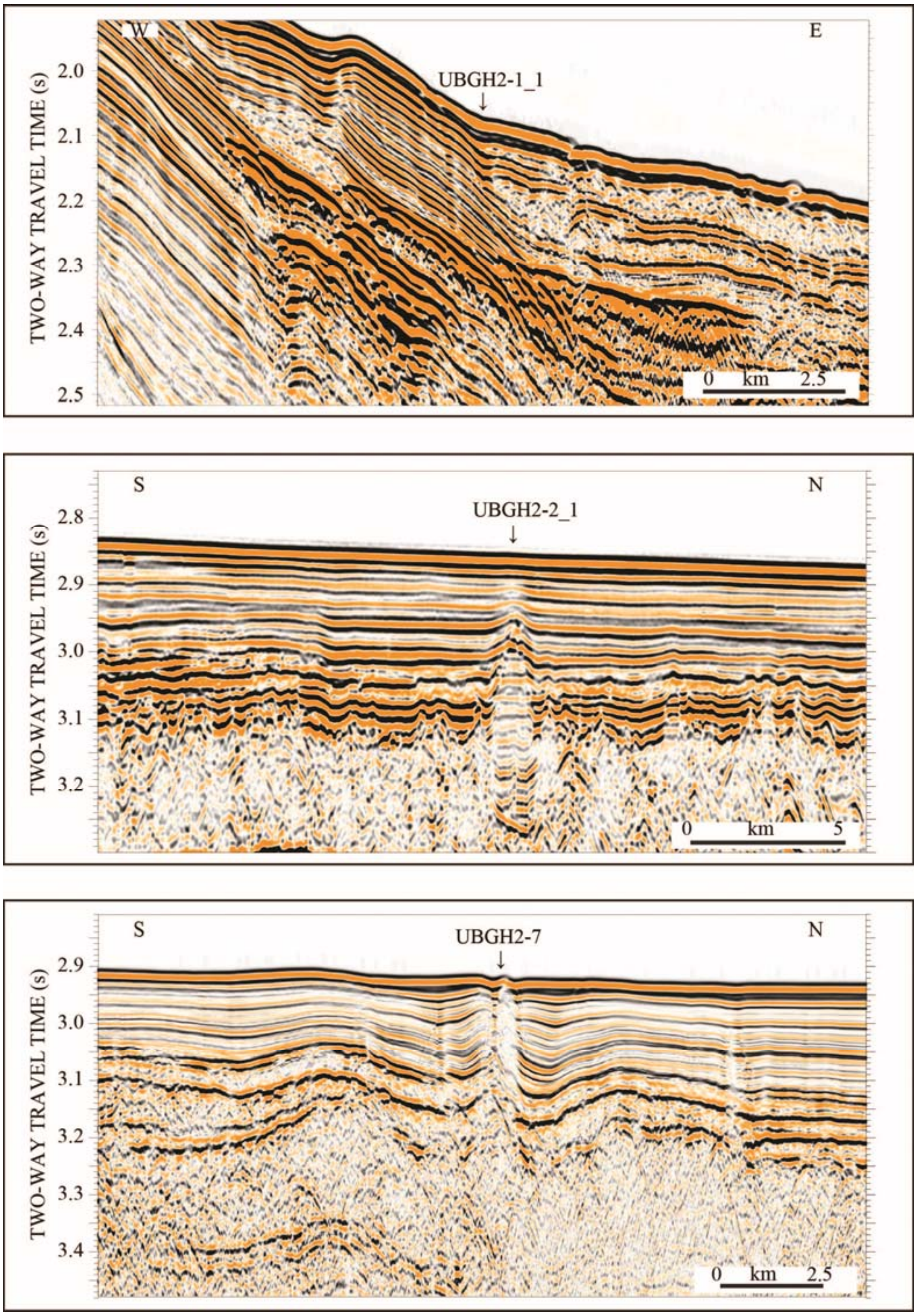

Fig. S1. Seismic profiles of non-chimney site UBGH2-1_1 (U1), truncated chimney site UBGH2-2_1 (U2), and chimney site UBGH2-7 (U7) in the Ulleung Basin (from literature)[6,33,36].

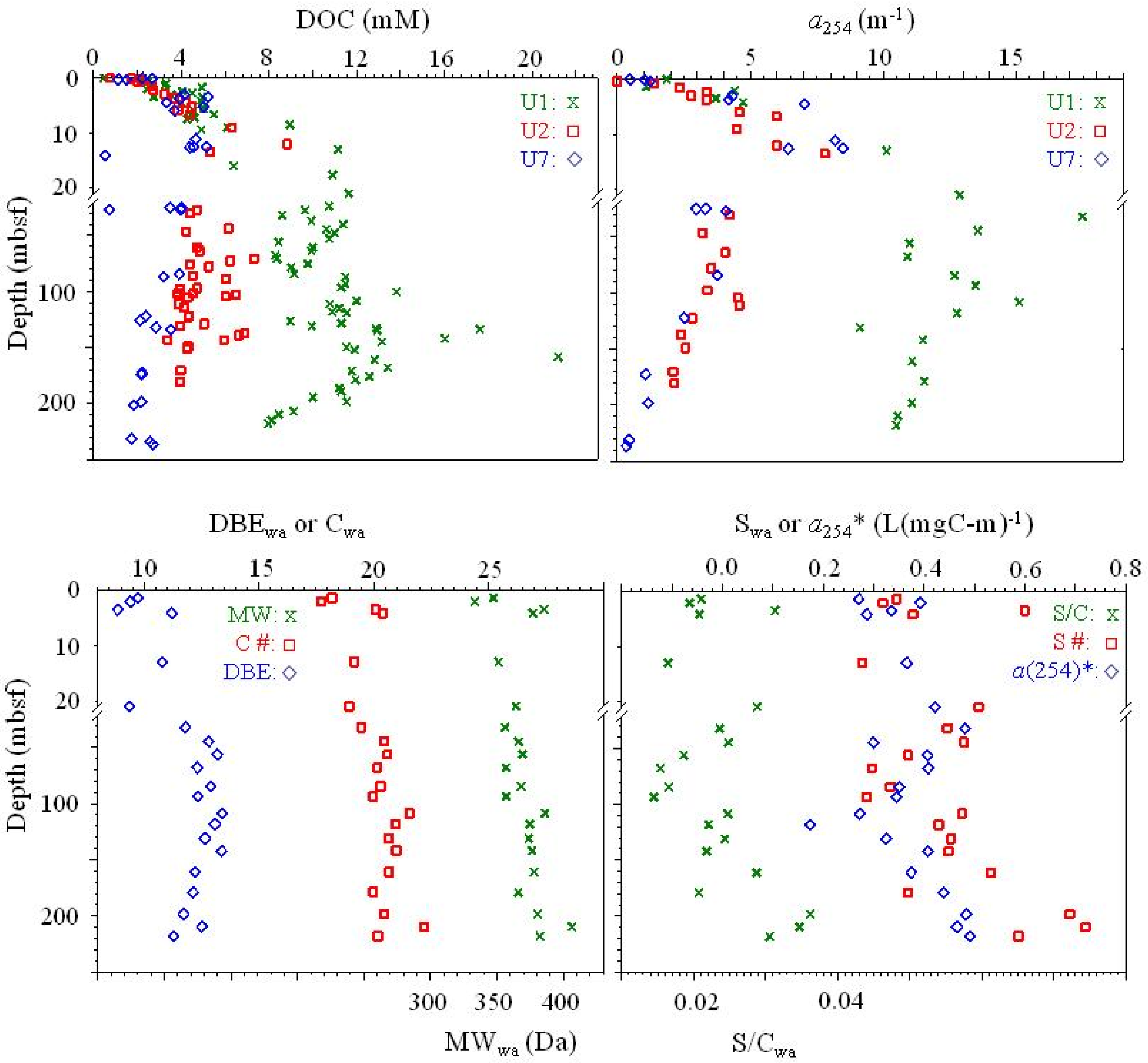

395

396 Fig. S2 Upper panel: Downcore profile of PWDOM parameters at sites U1, U2, and U7. Lower
397 panel: Downcore profile of AEOM parameters at site U1. Note the top 21 m is shown in
398 amplified scale.

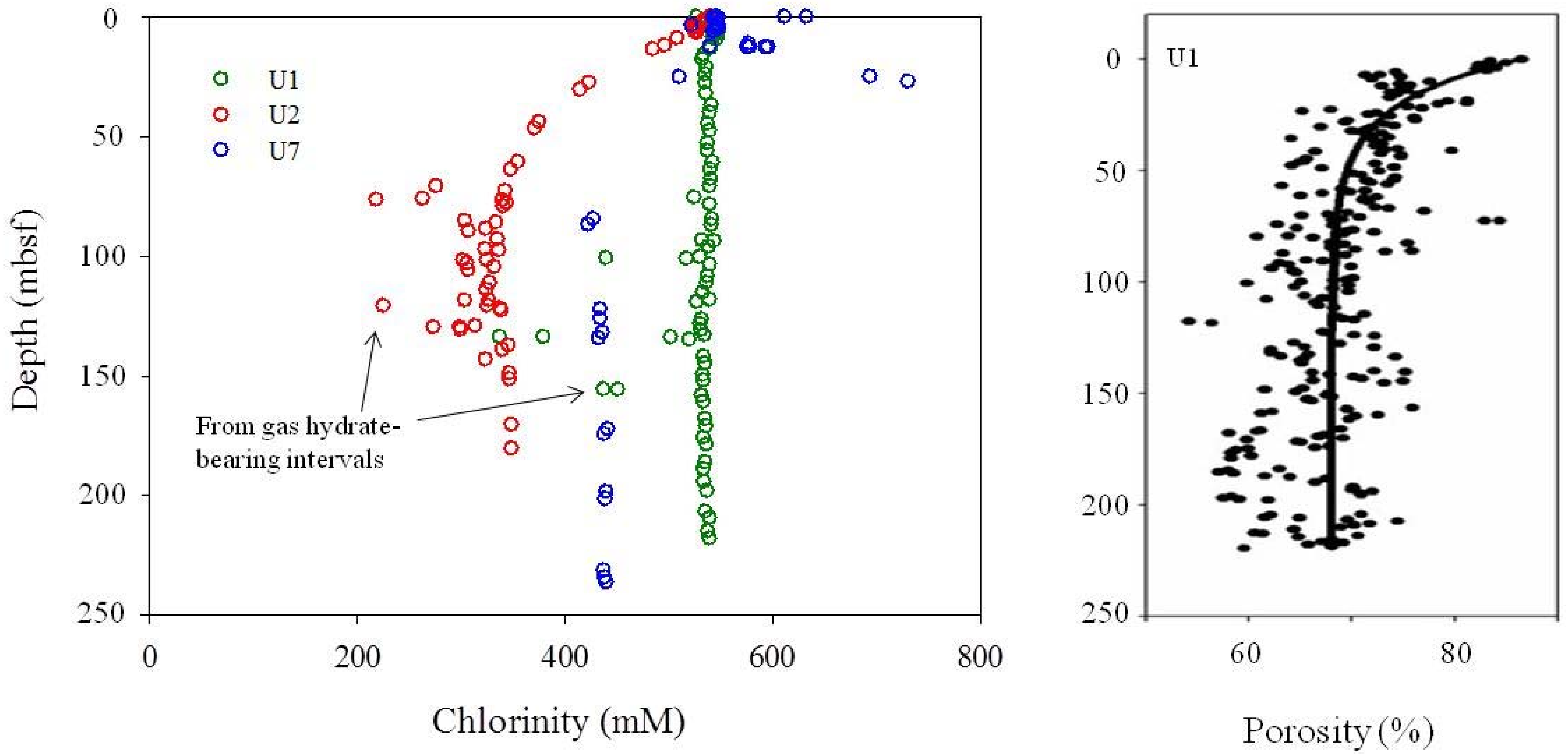

Fig. S3. Downcore profiles of chlorinity at three sites (left) and downcore profile of porosity at site U1 (right). Some left-shifted samples (smaller Cl^- concentration) are collected from gas hydrate-bearing intervals. Original data of chlorinity and porosity figure derived from literature[34,35].

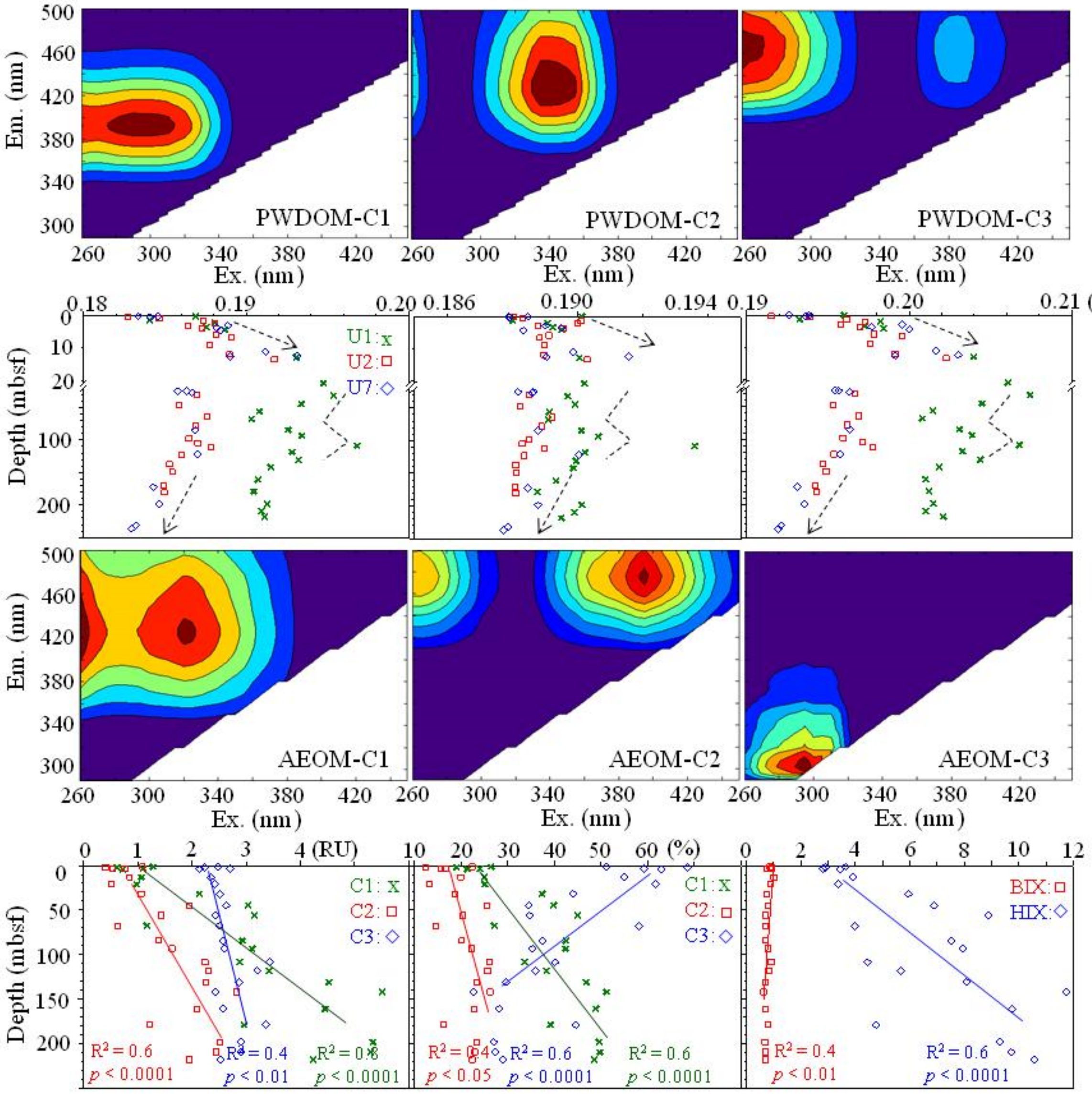

Fig. S4 Contour plots of identified EEM-PARAFAC components and downcore profiles of PWDOM (2nd row) and AEOM (4th row) proxies.

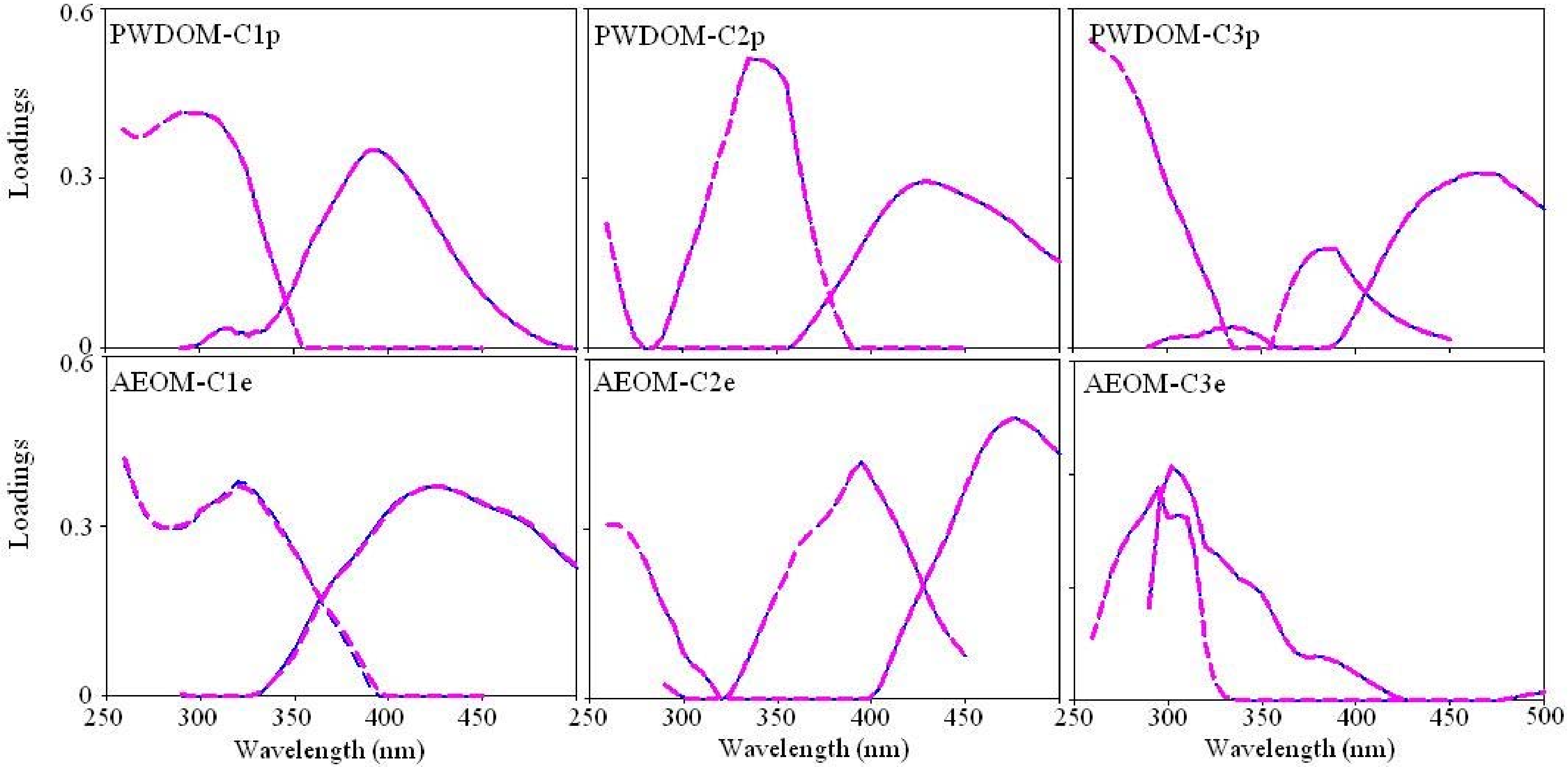

Fig. S5 Split-half validation of three identified EEM-PARAFAC components for PWDOM from sites U1, U2, and U7 (upper panel) and for AEOM from site U1 (lower panel).

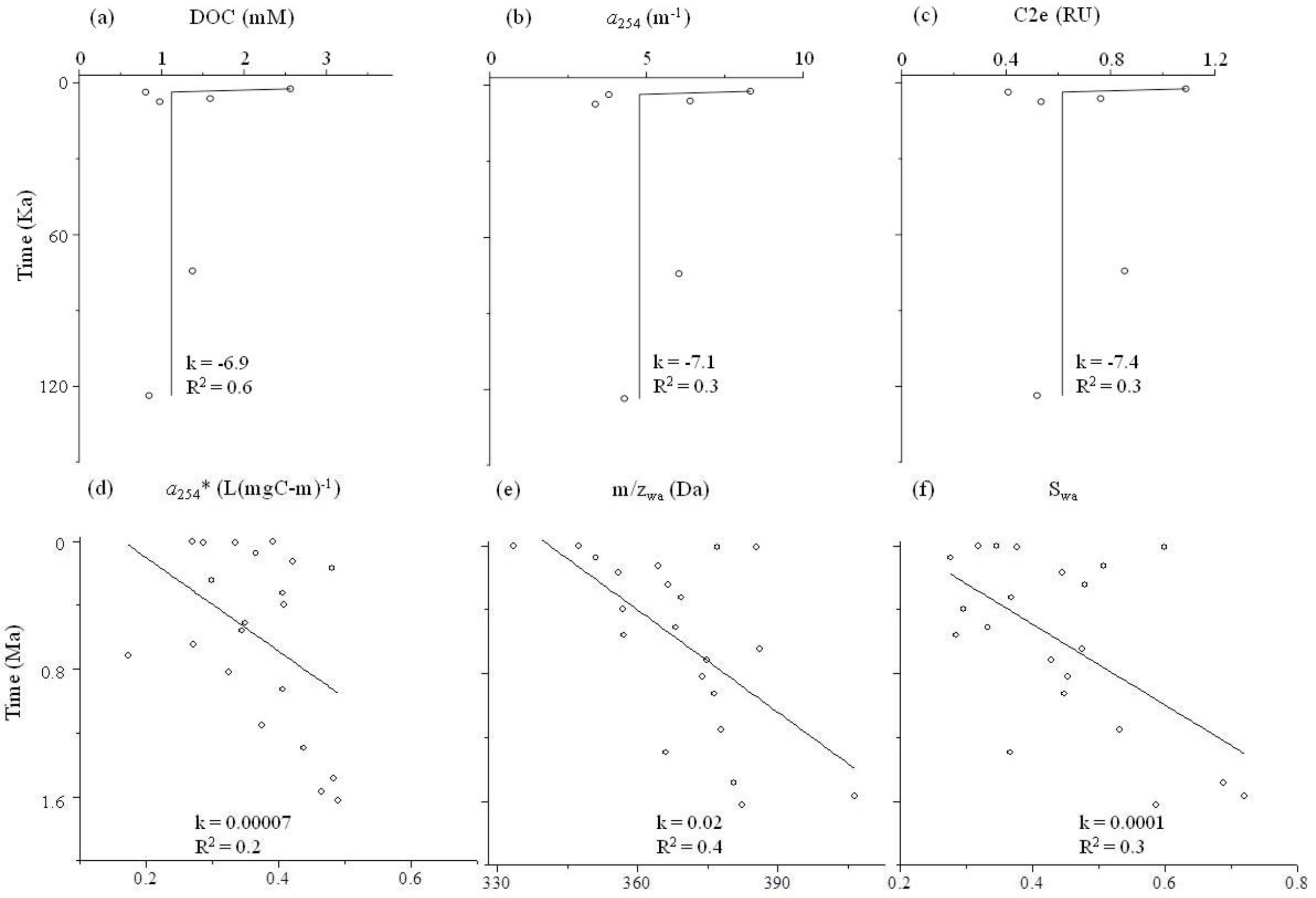

Fig. S6 Exponential decrease of DOC, CDOM, and FDOM above SMTZ (~21 mbsf) and linear increase of specific absorption coefficient a_{254}*, m/z_{wa}, and S_{wa} (humification) of AEOM with depth at site U1. k unit: Kyr^{-1}. $p < 0.05$.

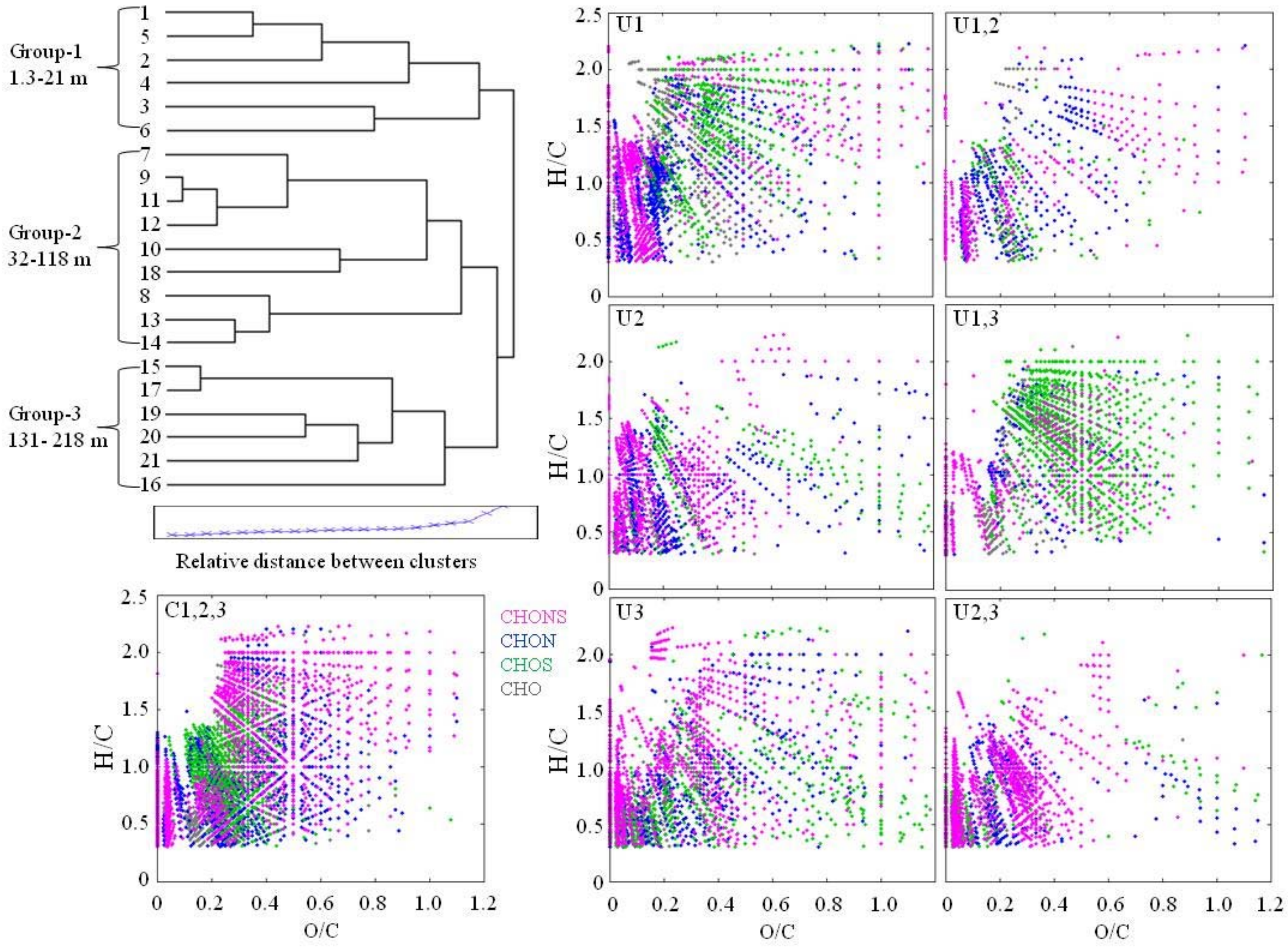

Fig. S7 Van Krevelen diagrams of unique (U) and common (C) molecular formula among three sub-groups (G1-3) classified by Cluster analysis for AEOM at site U1. Clusters match well with the depth, excluding a sample at 179 mbsf (~1.3-Ma). Cluster analysis based on Ward method of AEOM data of DOC, optical, molecular, and elemental parameters.

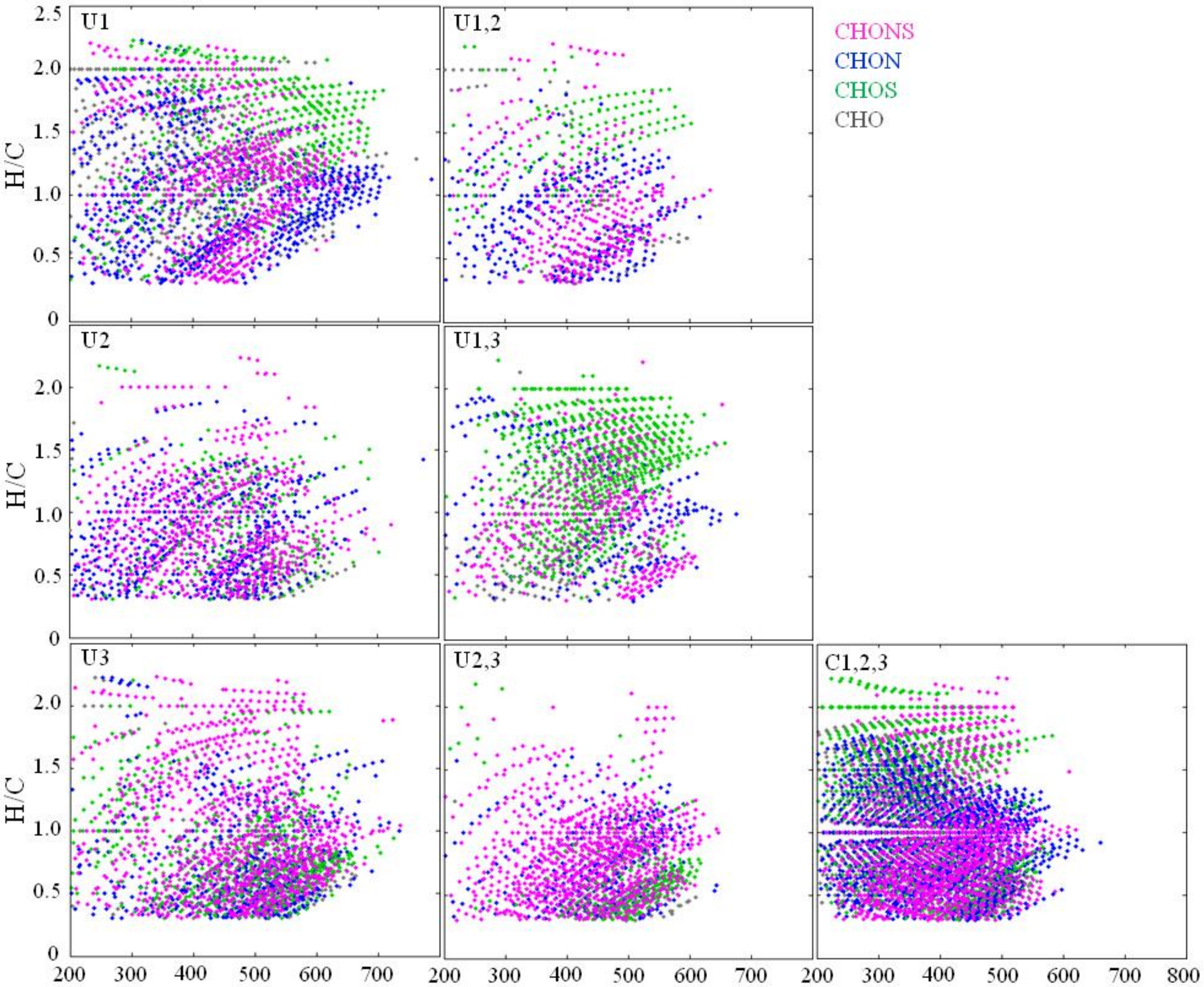

Fig. S8 H/C vs. m/z diagrams of unique (U) and common (C) molecular formula among three sub-groups (1-3) classified by cluster analysis for AEOM at site U1.

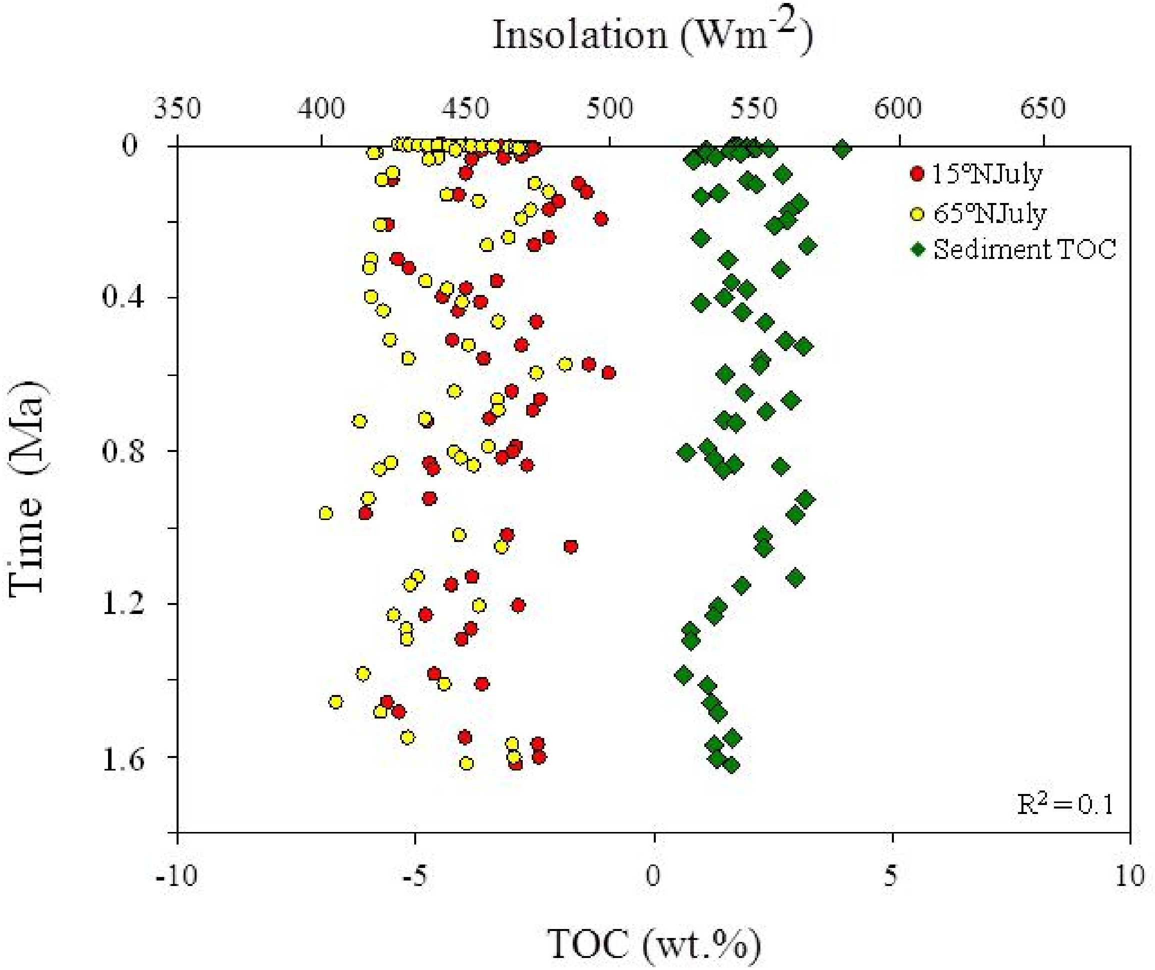

Fig. S9 Co-variation of the insolation at 15°N and 65°N in July with sediment total organic carbon (TOC) in a ~405-Kyr cycles at site U1. Linear correlation excluding the depth above extended SMTZ (21 mbsf) and below ~118 mbsf.

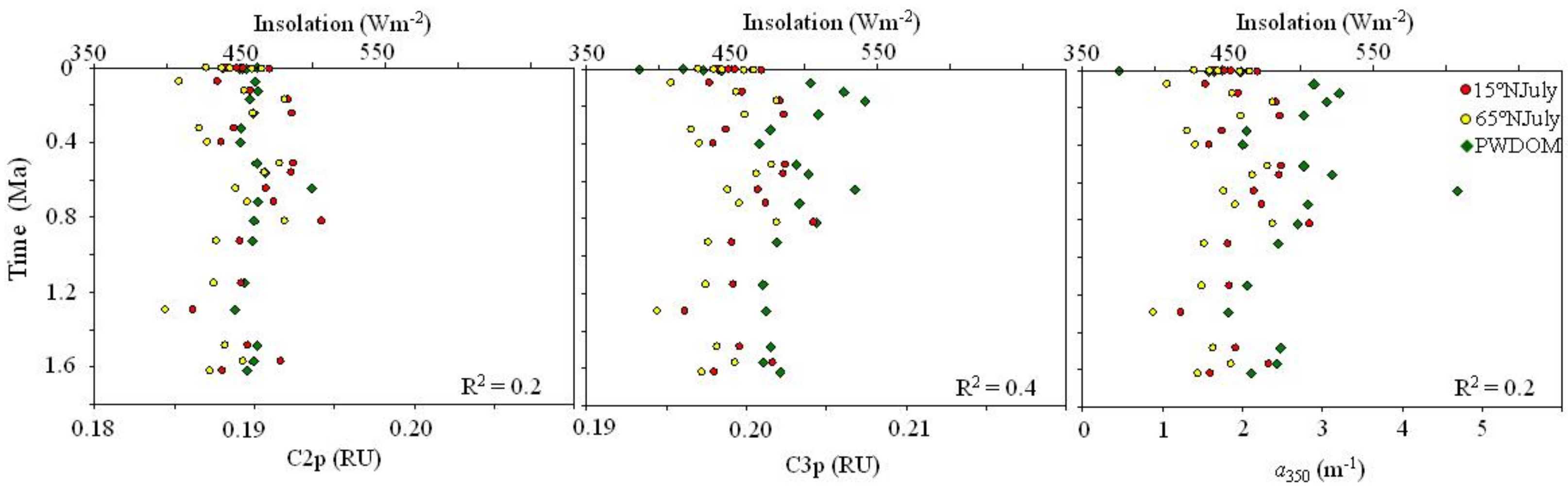

Fig. S10 Co-variation of the insolation at 15°N and 65°N in July with PWDOM parameters in a ~405-Kyr cycles at site U1. Linear correlation with depth ≤ 118 mbsf (718-Ka) and > 21 mbsf (123-Ka) ($p < 0.05$). Insolation data and the depth-age conversion at this site are based on literature[6,32].

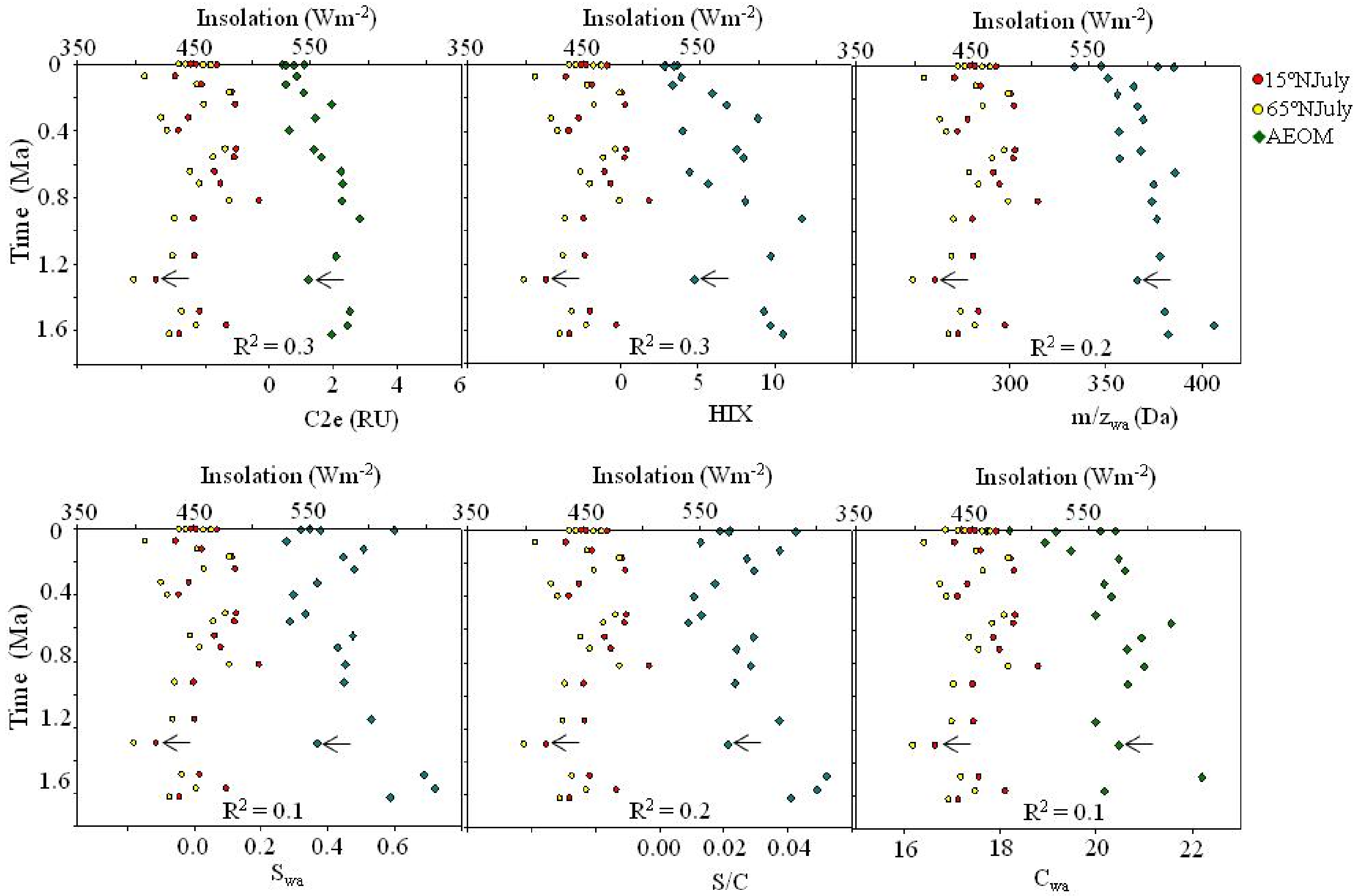

Fig. S11 Co-variation of the insolation at 15°N and 65°N in July with AEOM parameters in a ~405-Kyr cycles at site U1. Linear correlation with depth ≤ 118 mbsf (718-Ka) and > 21 mbsf (123-Ka). The arrows indicate the outlier depth at 179 mbsf (1.3-Ma) coincident with a very low insolation. Insolation data and the depth-age conversion at this site are based on literature[6,32].

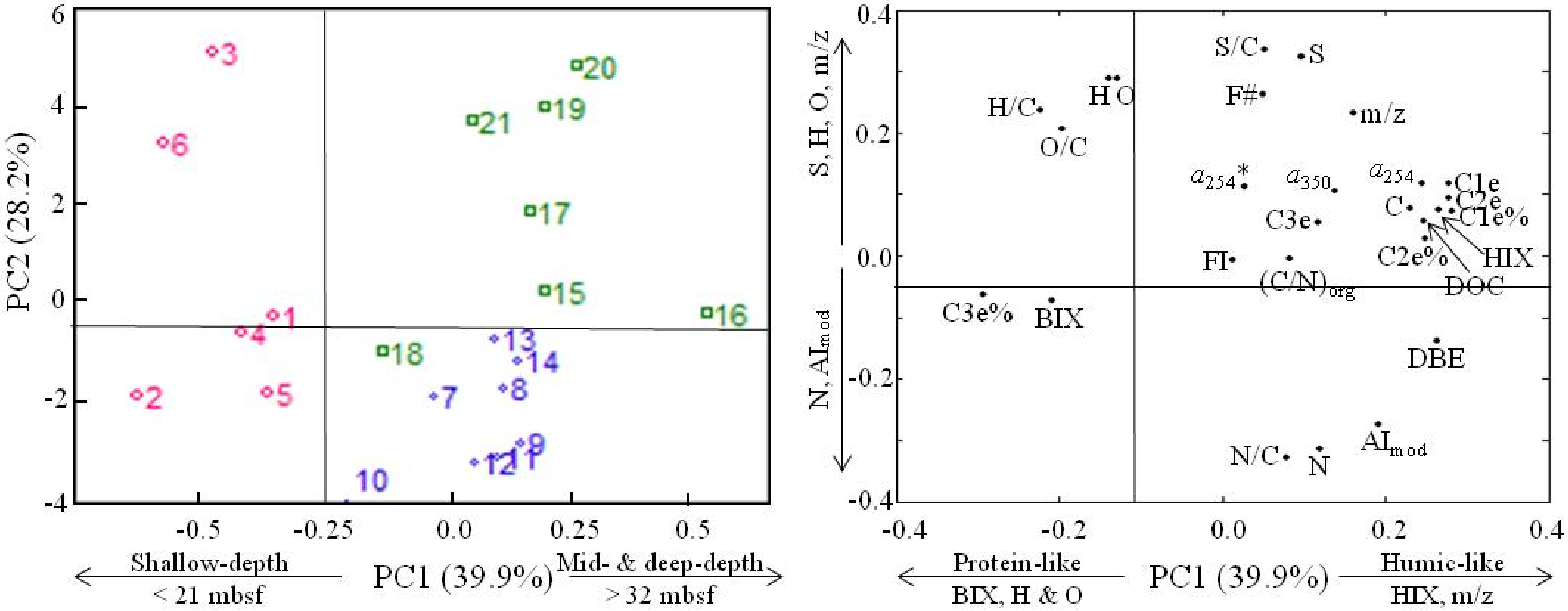

Fig. S12 Score plot (left) and loading plot (right) of principal component analysis (PCA) based on the DOM parameters of AEOM.

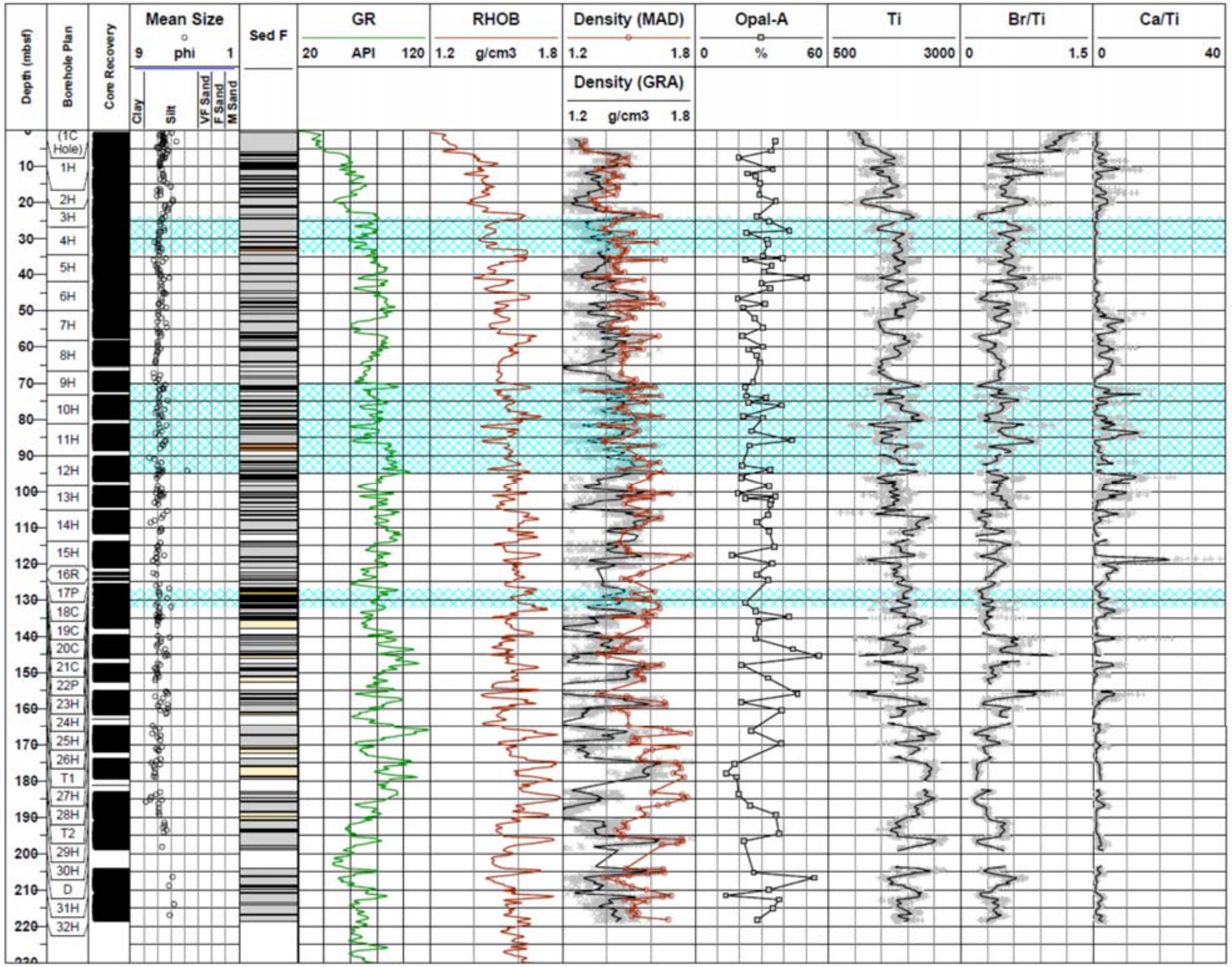

Fig. S13. Summary of distributions of sedimentary facies, core density, and other geological data from the site U1 (figure derived from literature)[6].

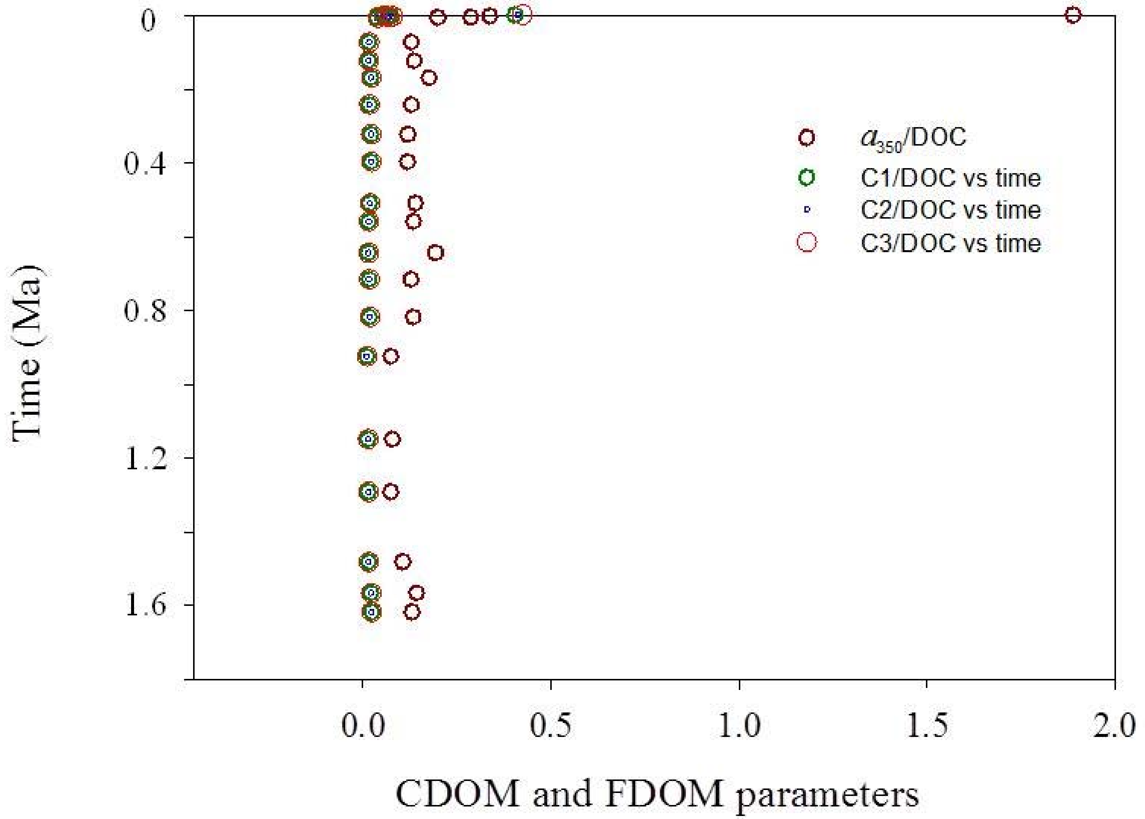

Fig. S14. Downcore profile of DOC-normalized CDOM and FDOM parameters of PWDOM.
